# A Comparison of Two Monoterpenoid Synthases Reveals Molecular Mechanisms Associated With the Difference of Bioactive Monoterpenoids Between *Amomum villosum* and *Amomum longiligulare*

**DOI:** 10.3389/fpls.2021.695551

**Published:** 2021-08-12

**Authors:** Haiying Zhao, Meng Li, Yuanyuan Zhao, Xiaojing Lin, Huilin Liang, Jieshu Wei, Wuke Wei, Dongming Ma, Zhongyu Zhou, Jinfen Yang

**Affiliations:** ^1^Key Laboratory of Chinese Medicinal Resource from Lingnan (Ministry of Education), Guangzhou University of Chinese Medicine, Guangzhou, China; ^2^School of Pharmacy, Guangzhou Xinhua University, Guangzhou, China; ^3^Key Laboratory of Plant Resources Conservation and Sustainable Utilization, Guangdong Provincial Key Laboratory of Applied Botany, South China Botanical Garden, Chinese Academy of Sciences, Guangzhou, China

**Keywords:** *Amomum villosum*, *Amomum longiligulare*, bornyl acetate, transcriptome, bornyl diphosphate synthase, linalool synthase

## Abstract

The fruits of *Amomum villosum* and *Amomum longiligulare* are both used medicinally as *Fructus Amomi* the famous traditional Chinese medicine, however, the medicinal quality of *A. villosum* is better than that of *A. longiligulare*. Volatile terpenoids in the seeds, especially bornyl acetate and borneol, are the medicinal components of *Fructus Amomi.* The volatile terpenoids and transcriptome of developing seeds of *A. villosum* and *A. longiligulare* were compared in this study. The result revealed that the bornyl acetate and borneol contents were higher in *A. villosum* than in *A. longiligulare*. Additionally, six terpenoid synthase genes (*AlTPS1–AlTPS6*) were screened from the transcriptome of *A. longiligulare*, and *AlTPS2* and *AlTPS3* were found to share 98 and 83% identity with *AvTPS2* and *AvBPPS* (bornyl diphosphate synthase) from *A. villosum*, respectively. BPPS is the key enzyme for the biosynthesis of borneol and bornyl acetate. Biochemical assays improved that AlTPS2 had an identical function to AvTPS2 as linalool synthase; however, AlTPS3 produced camphene as the major product and bornyl diphosphate (BPP) as the secondary product, whereas AvBPPS produced BPP as its major product. There was only one different amino acid between AlTPS3 (A496) and AvBPPS (G495) in their conserved motifs, and the site-directed mutation of A496G in DTE motif of AlTPS3 changed the major product from camphene to BPP. Molecular docking suggests that A496G mutation narrows the camphene-binding pocket and decreases the BPP-binding energy, thus increases the product BPP selectivity of enzyme. In addition, the expression level of *AvBPPS* was significantly higher than that of *AlTPS3* in seeds, which was consistent with the related-metabolites contents. This study provides insight into the TPS-related molecular bases for the biosynthesis and accumulation differences of the bioactive terpenoids between *A. villosum* and *A. longiligulare*. *BPPS*, the key gene involved in the biosynthesis of the active compound, was identified as a target gene that could be applied for the quality-related identification and breeding of *Fructus Amomi*.

## Introduction

*Amomum villosum* and *Amomum longiligulare* are plants of the genus *Amomum* (family *Zingiberacea*) that grow in Southeast Asia. According to the China Pharmacopoeia, both the dried and ripe fruits of *A. villosum* and *A. longiligulare* are used as the traditional Chinese medicine, *Fructus Amomi* (Sharen), which is used to treat digestive diseases such as abdominal pain, vomiting and dysentery (Commission of Chinese Materia Medica, 1999; Commission of Chinese Pharmacopoeia, 2015). In addition, *Fructus Amomi* is widely used in Chinese cuisine.

The main part of *Fructus Amomi* with medicinal value is its highly aromatic seeds, which contain rich essential oil. As the most active and abundant ingredient in the essential oil of seeds, bornyl acetate is the important medicinal component, and used as the quality standard of *Fructus Amomi* (Commission of Chinese Pharmacopoeia, 2015); in addition to its traditional effects (analgesia and antidiarrheic), bornyl acetate has been reported to have antioxidant, anti-inflammatory, antiabortion and anticancer activities (Kim et al., 2013; Chen et al., 2014; Yang et al., 2014; Li and Wang, 2016). However, the content of bornyl acetate in the seeds of *A. longiligulare* is lower than that of *A. villosum* (Qin et al., 2017). Because the medicinal quality of *A. villosum* is better than that of *A. longiligulare*, *A. villosum* is used more widely and has a higher economic value. Genetic identification and evaluation of these two species and other close species with similar morphological traits has been conducted using molecular identification based on DNA barcoding, single nucleotide polymorphisms (SNPs) in the ITS (internal transcribed spacer) and the chloroplast genome (Huang et al., 2014; Cui et al., 2019; Doh et al., 2019). However, metabolomic and transcriptomic comparisons of these species and the molecular mechanisms responsible for the differences in their photochemistry have not yet been investigated.

**FIGURE 1:**
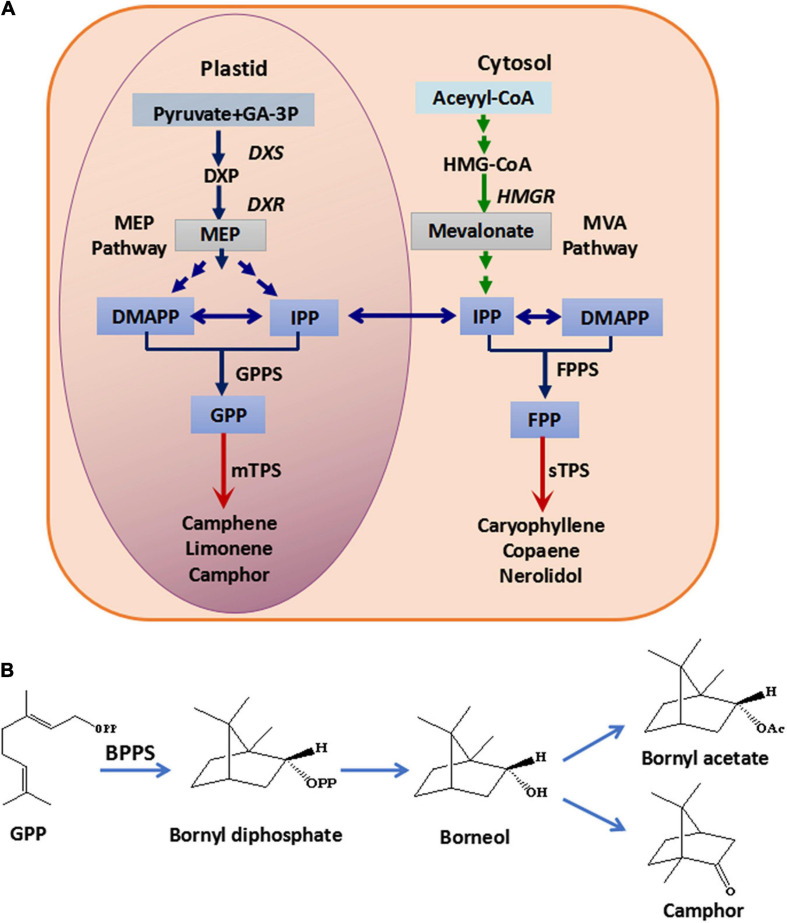
Monoterpenoid and sesquiterpenoid biosynthesis pathway **(A)** and the biosynthesis pathway from GPP to borneol, camphor and bornyl acetate **(B)**. GA-3P, glyceraldehyde-3-phosphate; HMG-CoA, 3-hydroxy-3-methylglutaryl coenzyme A; DXS, 1-deoxy-D-xylulose-5-phosphate synthase; DXP, 1-deoxy-D-xylulose-5-phosphate; DXR, 1-deoxy-D-xylulose-5-phosphate reductoisomerase; HMGR, 3-hydroxy-3-methyl glutaryl coenzyme A reductase; DMAPP, dimethylallyl diphosphate; IPP, isopentenyl diphosphate; MEP, 2-C-methyl-D-erythritol-4-phosphate; MVA, mevalonic acid; GPPS, geranyl diphosphate synthase; GPP, geranyl diphosphate; FPPS, farnesyl diphosphate synthase; FPP, farnesyl diphosphate; mTPS, monoterpenoid synthase; sTPS, sesquiterpenoid synthase; BPPS, bornyl diphosphate synthase.

The constituents of the essential oil in *Fructus Amomi* from *A. villosum* were identified, and the most abundant components were found to be monoterpenoids, such as bornyl acetate, borneol and camphor (Yu et al., 2005; De et al., 2011; Xue et al., 2015). In plants, terpenoids are synthesized by the mevalonate (MVA) pathway in the cytoplasm and the 2-C-methyl-D-erythritol-4-phosphate (MEP) pathway in the plastid, while terpenoid synthases (TPS) such as mono-TPS and sesqui-TPS catalyze geranyl diphosphate (GPP) and farnesyl diphosphate (FPP) to form diverse terpenoids (Tholl, 2015; Figure 1A). The genes encoding 1-deoxy-D-xylulose-5-phosphate reductoisomerase (DXR), 1-deoxy-D-xylulose-5-phosphate synthase (DXS) and 3-hydroxy-3-methylglutaryl Coenzyme A reductase (HMGR) for terpenoid backbone biosynthesis in *A. villosum* have been cloned and identified (Yang et al., 2012; Wang et al., 2014). Additionally, *AvBPPS* (bornyl diphosphate synthase) and AvPS (pinene synthase), which are involved in monoterpenoid biosynthesis in *A. villosum* have been cloned and functional characterized (Wang et al., 2018). BPPS catalyzes GPP to produce bornyl diphosphate (BPP), which is the precursor of borneol, and borneol is the precursor of bornyl acetate and camphor (Figure 1B). Therefore, BPPS is the key enzyme for the biosynthesis of borneol, bornyl acetate and camphor, which are the main monoterpenoids of *A. villosum* and *A. longiligulare*. Except for AvBPPS, BPPS genes have been identified from other plants, including *Salvia officinalis* (*SoBPPS*), *Lavandula angustifolia* (*LaBPPS*), *Lippia dulcis* (*LdBPPS*), and *Cinnamomum burmanni* (*CbTPS1*) (Wise et al., 1998; Despinasse et al., 2017; Hurd et al., 2017; Ma et al., 2021). However, the enzymes/genes, especially the key enzyme BPPS, involved in the pathway of the main bioactive components in *A. longiligulare* remain uninvestigated.

As we mentioned above, terpenoids including bornyl acetate, borneol, are the bioactive compounds of *A. villosum* and *A. longiligulare*, knowledge about terpenoids and their biosynthesis of *A. longiligulare* and comparisons of *A. villosum* and *A. longiligulare* can facilitate increased understanding of the biological foundation for differences in their quality. Furthermore, exploring the genes involved in the volatile terpenoid biosynthesis in *A. longiligulare* and *A. villosum* will provide insight into the evolution of these two species and lead to improved bioactive terpenoid production.

In recent years, integration of metabolomics and transcriptomics has been utilized to investigate terpenoid biosynthesis in plants (Wei et al., 2016; Chen et al., 2018; Torrens-Spence et al., 2018). Based on our previous report that the *AvBPPS* gene was expressed at the highest levels in the seeds of 45 days after flowering (DAF) of *A. villosum* (Wang et al., 2018), the 45-DAF seeds of *A. longiligulare* were used for transcriptomics and metabolomics analysis in this study. The transcriptomics data from *A. villosum* and *A. longiligulare* were then used to compare the genes involved in the terpenoids biosynthesis and mine TPS genes. *AlTPS2* and *AvTPS2* sharing high identity were cloned and characterized. *AlTPS3* had high identity with *AvBPPS*, then was cloned and characterized as well. The comparisons based on the sequence, enzymatic function and expression of these two couples of TPS genes, especially *AlTPS3* and *AvBPPS*, provided insight into the differences in terpenoids biosynthesis in *A. villosum* and *A. longiligulare*.

## Materials and Methods

### Plant Materials

The flowers and 45-DAF fruits from *A. longiligulare* and *A. villosum* were collected and frozen at −80°C. The fruit was separated into pericarps and seeds. In addition, the mature fruits (approximately 90-DAF) of *A. villosum* and *A. longiligulare* were dried to a constant weight at 50°C and then stored in a dryer.

### Volatile Terpenoid Extraction and Analysis

Approximately 0.3 g of fresh or dry materials was ground frozen in liquid nitrogen, vortexed and then extracted with 1.5 mL hexane using an ultrasonic cleaner for 0.5 h followed by incubation at 40°C for 1 h. Samples were then centrifuged at 12,000 rpm for 5 min, after which the resulting supernatants were pipetted into new 2 mL tubes. Next, 1 mL of hexane extract was pipetted into 1.5 mL vials for GC-MS analysis. The extracts were then analyzed using an Agilent 7890B Gas Chromatograph with a 5977B inert Mass Selective Detector (Agilent, United States). Helium was used as the carrier gas (1 mL/min), then separated on an HP-5MS column (30 m × 250 μm × 0.25 μm film thickness). For separation, the GC oven temperature was programmed for an initial temperature of 35°C for 5 min followed by an increase of 12°C/min to 300°C, which was held for 5 min. A NIST17/demo1 Mass Spectral Library was used for metabolite identification. The terpenoid compounds were identified by the mass spectral library, after which the main monoterpenoids investigated in this study, including α-pinene, camphene, myrcene, limonene, linalool, camphor, borneol and bornyl acetate, were further identified using their authentic standards. The contents were quantified based on bornyl acetate standard curves, and there were three biological replicates for each tissue.

### RNA Extraction

The total RNA of each sample (including seeds, pericarps and flowers) was isolated using an OmniPlant RNA kit (CWbiotech, China), following the manufacturer’s protocol. For the RNA extraction of seeds, the following step was added to remove the polysaccharides: after blending by vortexing the lysate and sample, 1/20 volume of isopropyl alcohol and 1/20 volume of high salt solution mixture (1.2 M NaCl + 0.8 M sodium citrate) were added to the lysate and blended upside down. The RNA quality was then verified using an ultraviolet spectrophotometer (TIANGEN, China) and checked using RNase free agarose gel electrophoresis. RNA with an OD260/OD280 of 1.8–2.2 was used for subsequent analyses.

### Transcriptome Sequencing, *de novo* Assembly and Annotation

RNA extracted from the 45-DAF seeds was used for the transcriptome sequencing without replicates. The library preparation and transcriptome sequencing, *de novo* assembly and annotation were conducted by Gene *De novo* Co., as described previously (He et al., 2018; Wang et al., 2018).

### Screening of Candidate AlTPS Genes

The unigenes of candidate AlTPS genes were screened out from the Pfam annotation (Finn et al., 2016) of the transcriptome data of *A. longiligulare* using the keyword “terpene synthase,” combining with the local-Blast using the sequences of AvTPS1–AvTPS10 (Wang et al., 2018). The unigenes shorter than 500 bp were filtered out.

### Cloning of the Full-Length Coding Region of *AlTPS2*, *AvTPS2*, and *AlTPS3*

The gene-specific primers used for *AlTPS2*, *AvTPS2*, and *AlTPS3* cloning are listed in Supplementary Table 1. Because *AvTPS2* lacks a complete ORF (open reading frame), its 5′-end and 3′-end were amplified using a SMARTer^®^ RACE Kit (Takara, Japan) according to the user manual. The full-length cDNA was amplified using Prime STAR Max DNA Polymerase (Takara, Japan) following the manufacturer’s instructions. The PCR conditions used were as follows: 98°C for 1 min followed by 30 cycles of 98°C for 10 s, 50–60°C for 15 s, and 72°C for 15 s, and then final extension at 72°C for 5 min. The product was then ligated into pLB cloning vector (Tiangen, China), which was subsequently transformed into *Escherichia coli* DH5α and sequenced.

### Prokaryotic Expression and Protein Purification of AlTPS3, AlTPS2, and AvTPS2

ORFs of *AlTPS3, AlTPS2*, and *AvTPS2* excluding the N-terminal transit peptides were amplified, and proper restriction enzyme sites were added at each end by PCR using the primers described in Supplementary Table 1. The ORFs were then ligated into the pET32a expression vector for *AvTPS2* and *AlTPS3* and the pMAL-c5X vector for *AlTPS2* using an In-Fusion Cloning Kit (Takara, Japan). Next, the positive constructs were transformed into competent Rosetta (DE3) cells, and the positive colonies were inoculated into LB media containing 50 μg/mL carbenicillin and 25 μg/mL chloramphenicol and incubated at 37°C until the OD 600 reached 0.4–0.6. The proteins were subsequently induced by incubation in the presence of 0.1 mM isopropyl-β-D-1-thiogalactopyranoside (IPTG) at 16°C for 16 h. Next, the recombined proteins were purified with NI-NTA resin (Qiagen, Hilden, Germany) or MBP-tag columns (Qiagen, Smart-Life Sciences, China), following the relative manual recommendations: for NI-NTA resin column, unbound proteins were washed with 20 mM imidazole phosphate buffer (pH 7.4) and then target proteins (AvTPS2 and AlTPS3) were eluted by 200 mM imidazole phosphate buffer; for MBP-tag columns, unbound protein was washed by eluant A (20 mM Tris-HCl, 200 mM NaCl, 1 mM EDTA and DTT, pH 7.4) and then target protein (AlTPS2) was collected using eluant B (eluant A adding 20 mM maltose). The purified proteins were dialyzed in PD-10 Desalting Columns (GE Healthcare).

### Enzyme Assay and Product Analysis

The enzymatic reaction conditions and methods for the analysis of AlTPS2, AvTPS2, and AlTPS3 were conducted as described by Wang et al. (2018). The enzyme reacted with the substrate GPP for 1 h at 30°C, after which samples were dephosphorized by treatment with 1.0 μL alkaline phosphatase (Thermo Fisher, United States) for another hour at 37°C with 200 μL hexane overlaid. The hexane phase was then extracted and used for GC-MS analysis. Enzymatic products were detected based on GC-MS as previously described (Wang et al., 2018). The NIST17 Mass Spectral Library was used for metabolite identification. Additionally, the standards of linalool, camphene, limonene and borneol were utilized for further identification. Each enzyme assay was performed with at least three replicates.

### Construction of AlTPS3-A496G and AvBPPS-G495A Mutants and Enzyme Assay

The primers for site-directed mutation were listed in Supplementary Table 1. The mutant of AlTPS3-A496G and AvBPPS-G495A were performed using a site-directed mutagenesis kit (TIANGEN, Beijing, China). PCR mixture in a volume of 50 μL consisted of 1 U of FastAlteration DNA Polymerase, 5 × FastAlteration Buffer, 10 μM primers, and 50 ng of recombinant plasmid pET-32a -AlTPS3. The PCR mixture was denatured at 95°C for 2 min, followed by 30 cycles of 20 s at 94°C, 10 s at 62°C, and 2.5 min at 68°C, and a final 5-min extension at 72°C, the PCR product was incubated at 37°C with *Dpn*I restriction enzyme for 1 h to remove the template plasmid. Then, the PCR product was transformed into competent cells of *E. coli*, and the mutant site was confirmed by sequencing. The subsequent prokaryotic expression, protein purification were performed following the methods mentioned above. Enzyme assay was performed to compare the products of wild type and mutant using the methods described above with the overnight incubation with GPP instead of 1 h.

### Molecular Modeling and Docking

The secondary structures of the proteins were predicted through SwissModel Workspace,^1^ using 1N24.1.A [crystal structure of (+) -bornyl diphosphate synthase] protein as a template for homology modeling. The reliability of protein models was evaluated by Procheck^2^ and the energy was minimized by SPDBV. The secondary structures of GPP and BPP were downloaded from ZINC.^3^ The molecular docking was performed using AutoDock vina with grid points and spacing set as 50 × 50 × 50 and 0.375 Å (Trott and Olson, 2010). The resulting complexes were visualized with PyMOL.

### Quantitative Real-Time PCR

Quantitative real-time PCR (qRT-PCR) was performed to quantify the gene transcriptional expression. The primers (qRT-TPS2F/R and qRT-TPS3F/R) used for qRT-PCR are shown in Supplementary Table 1. qRT-PCR was conducted using 2 × M5 HiPer SYBR Premix EsTaq (Mei5 Biotechnology, China) on a CFX 96 Real-Time PCR Detection System (BioRad, United States) following the manufacturers’ instructions. The thermal cycling conditions were 95°C for 10 s, followed by 40 cycles of 95°C for 5 s and 58°C for 30 s. During the reaction, the target gene transcript levels were monitored using a reference gene and calculated using the 2^–△△Ct^ method. All experiments were performed with three biological replicates and three technical replicates.

### Bioinformatics Analysis

Multi-sequence alignment was conducted using the DNAMAN software. A phylogenetic tree was constructed by the neighbor-joining method using the MEGA-X software, after which the constructed phylogenetic tree was imported into the iTOL^4^ for modification. Plastid transport peptide was analyzed using the ChloroP 1.1 Server.^5^

## Results

### Comparison of Volatile Terpenoids in Seeds of *A. longiligulare* and *A. villosum*

**FIGURE 2:**
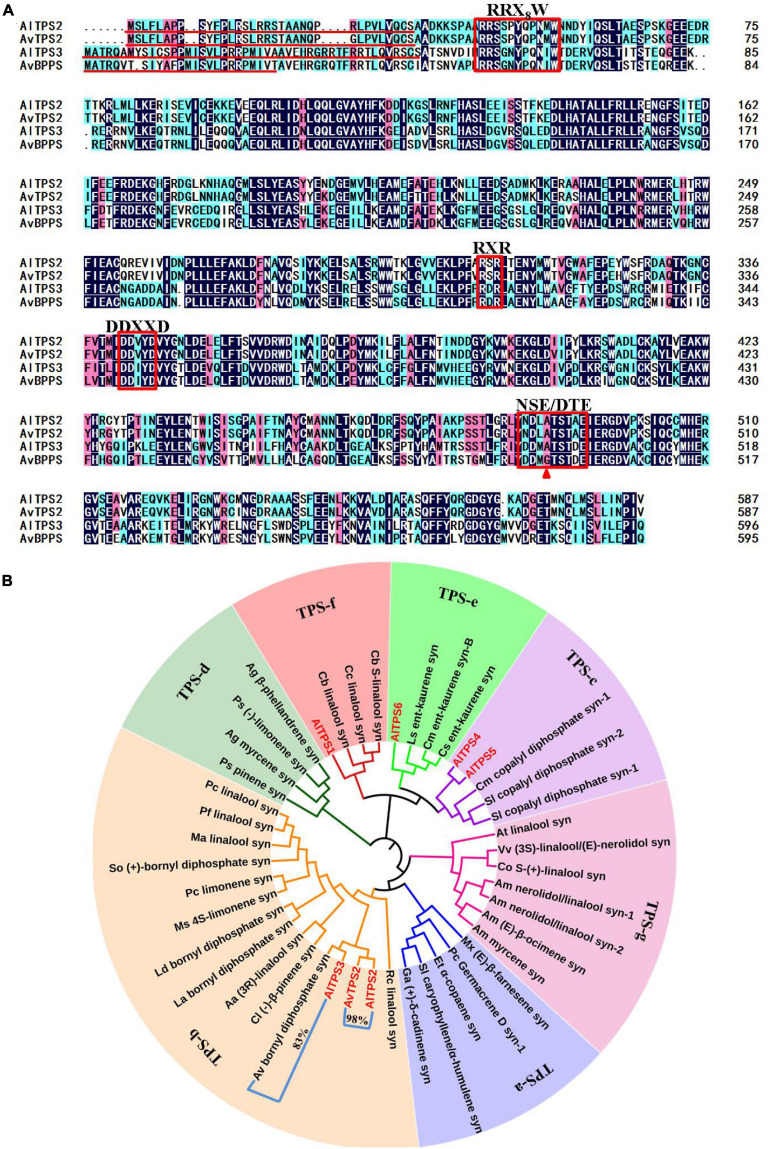
Amino acid sequence alignment of AlTPS2, AlTPS3, AvTPS2 and AvBPPS **(A)** and phylogenetic analysis of AlTPSs from *A. longiligulare*, AvTPS2 and terpenoid synthases from other plants **(B)**. The red underline indicates the plastid transport peptide. The red box represents the conserved motifs. The red triangle indicates the different amino acids between AlTPS3 and AvBPPS. The number in **(B)** represents the identity of the amino acid sequences of two genes clustered into one branch.

Seeds are the main medicinal part of *Fructus Amomi*, which are abundant in volatile terpenoids, therefore, the volatile terpenoids in 45-DAF seeds of *A. longiligulare* and *A. villosum* were analyzed by GC-MS. In total, 21 monoterpenoids and 16 sesquiterpenoids were detected in *A. longiligulare*, while 20 monoterpenoids and 21 sesquiterpenoids were detected in *A. villosum*, and both the total contents of monoterpenoids and sesquiterpenoids of *A. villosum* are higher than those of *A. longiligulare* (Supplementary Table 2). There were nine monoterpenoids only detected in *A. longiligulare* or *A. villosum*, for example linalool was only detected in *A. longiligulare*, while γ-terpineol was only detected in *A. villosum.* Monoterpenoids are the majority terpenoids in the seeds, and the percentages of main monoterpenoids, including bornyl acetate, bornel, camphor, D-limonene, myrcene, camphene and α-pinene, among the total volatile terpenoids are 50 and 56% in *A. longiligulare* and *A. villosum*, respectively (Supplementary Figures 1A,B). Specifically, the percentage of bornyl acetate of *A. villosum* is higher than that of *A. longiligulare*, however, the percentage of camphor of *A. longiligulare* is higher than that of *A. villosum* (Supplementary Figures 1C,D). Additionally, considering the mature and dried fruits are used as traditional medicine, the main monoterpenoids of mature and dried seeds of *A. longiligulare* and *A. villosum* were analyzed as well, and compared with the data of developing seeds. In mature seeds, bornyl acetate and camphor are the most abundant monoterpenoid in *A. villosum* and *A. longiligulare*, respectively (Supplementary Table 3). The contents of bornyl acetate and borneol of *A. villosum* are higher than those of *A. longiligulare*, but the content of camphor of *A. longiligulare* is higher than that of *A. villosum*. Camphor, borneol and bornyl acetate are produced from the same precursor BPP (Figure 2B); the total contents of these BPP-related terpenoids are higher in *A. villosum* than in *A. longiligulare* in spite of 45-DAF seeds or mature seeds (Table 1).

**TABLE 1:** Comparison of main monoterpenoid contents (μg/mg) in seeds of *A. logiligulare* and *A. villosum.*

Compound	Formula	45-DAF seeds (fresh)		Mature seeds (dry)	
		*A. longiligular*	*A. villosum*	*A. longiligular*	*A. villosum*
α-Pinene	C_1__0_H_16_	0.67 ± 0.04	1.75 ± 0.04	0.88 ± 0.08	1.63 ± 0.19
Camphene	C_1__0_H_16_	1.73 ± 0.23	3.13 ± 0.06	4.31 ± 0.58	4.85 ± 0.61
Myrcene	C_1__0_H_16_	1.33 ± 0.16	1.21 ± 0.02	0.73 ± 0.07	2.04 ± 0.26
D-Limonene	C_1__0_H_16_	1.81 ± 0.22	2.84 ± 0.04	2.03 ± 0.21	3.74 ± 0.49
Camphor	C_1__0_H_1__6_O	2.45 ± 0.28	1.35 ± 0.03	16.25 ± 1.20	8.70 ± 0.24
Borneol	C_1__0_H_1__8_O	0.85 ± 0.08	1.25 ± 0.03	1.83 ± 1.36	3.04 ± 0.38
Bornyl acetate	C_1__2_H_2__0_O_2_	5.23 ± 0.54	9.52 ± 0.11	7.47 ± 0.86	16.52 ± 1.00
BPP-related terpenoids*	−	8.53 ± 0.90	12.13 ± 0.61	25.55 ± 2.42	28.25 ± 1.61

*BPP-related terpenoisd means the terpenoids produced from BPP (bornyl diphosphate), including camphor, borneol and bornyl acetate.

### Transcriptomics Analysis and Comparison of Genes Involved in Terpenoid Backbone Biosynthesis Between *A. longiligulare* and *A. villosum*

In order to mine the BPPS gene and to explore the other genes involved in the terpenoid biosynthesis of *A. longiligulare*, the 45-DAF seeds were used for transcriptome sequencing. High-throughput sequencing and *de novo* assembly yielded 62,433 unigenes, and 36,746 unigenes (58.86%) were totally annotated using the major public databases Nr, KOG, Swiss-Prot and KEGG (Supplementary Table 4 and Supplementary Figure 2). The transcriptome data were submitted to the NCBI and assigned the SRA accession number SRR10769494. Based on the KEGG annotation, 1,527 unigenes were mapped to the biosynthesis of secondary metabolites, and 88 unigenes were annotated to terpenoid backbone biosynthesis. The KEGG annotation data of the 45-DAF seeds transcriptome of *A. villosum* (Wang et al., 2018) were used to compare the genes involved in the upstream pathway of terpenoid biosynthesis with *A. longiligulare*. The results revealed that all genes involved in the MVA and MEP pathway were annotated in both *A. longiligulare* and *A. villosum* transcriptome data, but more unigenes were annotated in total in *A. villosum* (62) than in *A. longiligulare* (58) (Supplementary Table 5). The identities of relative genes of *A. villosum* and *A. longiligulare* were all higher than 80%.

### Six AlTPS Candidate Genes Were Screened Out From the Transcriptome, and *AlTPS3*, *AlTPS2*, and *AvTPS2* Were Cloned

Based on the Pfam annotation of the transcriptome data of *A. longiligulare* and the local-Blast result *via* AvTPSs (Wang et al., 2018), six *AlTPS* unigenes with longer length were screened out (Supplementary Table 6). Of these genes, three (*AlTPS1-AlTPS3*) were predicted to encode the monoterpenoid synthase gene, and *AlTPS3* presented the highest RPKM expression value. According to the nucleotide sequence alignment, *AlTPS3* had the highest (95%) identity with *AvBPPS*, which has been identified as bornyl diphosphate synthase from *A. villosum* (Wang et al., 2018). *AlTPS2* shared 99% identity with *AvTPS2*, which had been previously screened from the transcriptome of *A. villosum* but yet cloned (Wang et al., 2018). *AlTPS2*, *AvTPS2* and *AlTPS3* were cloned in this work. The complete ORFs of both *AlTPS2* and *AvTPS2* were 1,764 bp, encoding 587 amino acids with a predicted 34-amino-acid plastid transit peptide on the N-terminal (Figure 2A). The complete ORF of *AlTPS3* was 1,791 bp, and it encoded 597 amino acids with a predicted 47-amino-acid transit peptide on its N-terminal, however, *AvBPPS* encoded 596 amino acids and its predicted transit peptide was shorter than *AlTPS3* (Figure 2A). The gene and deduced amino acid sequences of *AlTPS2*, *AvTPS2*, and *AlTPS3* have been submitted to GenBank under the accession numbers MN829548, MN829551, and MN829549, respectively.

The deduced amino acid sequences of six candidate AlTPSs and AvTPS2 (Supplementary Table 7), and other functional TPS identified from subfamilies a–g (Supplementary Table 8) were used to construct phylogenetic trees (Figure 2B). AlTPS2, AvTPS2, and AlTPS3 were clustered into the TPS-b subfamily, which is composed of monoterpenoid synthases. AlTPS1 was clustered into the TPS-f subfamily, while AlTPS4 and AlTPS5 were clustered into the TPS-c subfamily, and AlTPS6 was clustered into the TPS-e subfamily. The terpenoid synthases in the TPS-c and TPS-e subfamilies were found to be diterpenoid synthases. Moreover, AlTPS2 and AvTPS2 were clustered into one branch, while AlTPS3 and AvBPPS were clustered into another branch. Alignment of AlTPS2, AvTPS2, AlTPS3, and AvBPPS showed that they all contained the conserved mono-TPS motif, RRX_8_W, RXR, DDXXD, and NSE/DTE (Figure 2A). Comparison of the amino acid sequences revealed that the identity of AlTPS2 and AvTPS2 was 98%. The four conserved motifs of AlTPS2 and AvTPS2 were completely consistent, and their other amino acid regions were highly conserved as well (Supplementary Figure 3). However, the amino acid identity of AlTPS3 and AvBPPS was only 83%, although they were clustered into a close branch (Figure 2B). Therefore, we speculated that the functions of AvTPS2 and AlTPS2 might be the same, while the function of AlTPS3 might be very similar to AvBPPS.

### AlTPS2 and AvTPS2 Were Characterized as Linalool Synthase

**FIGURE 3:**
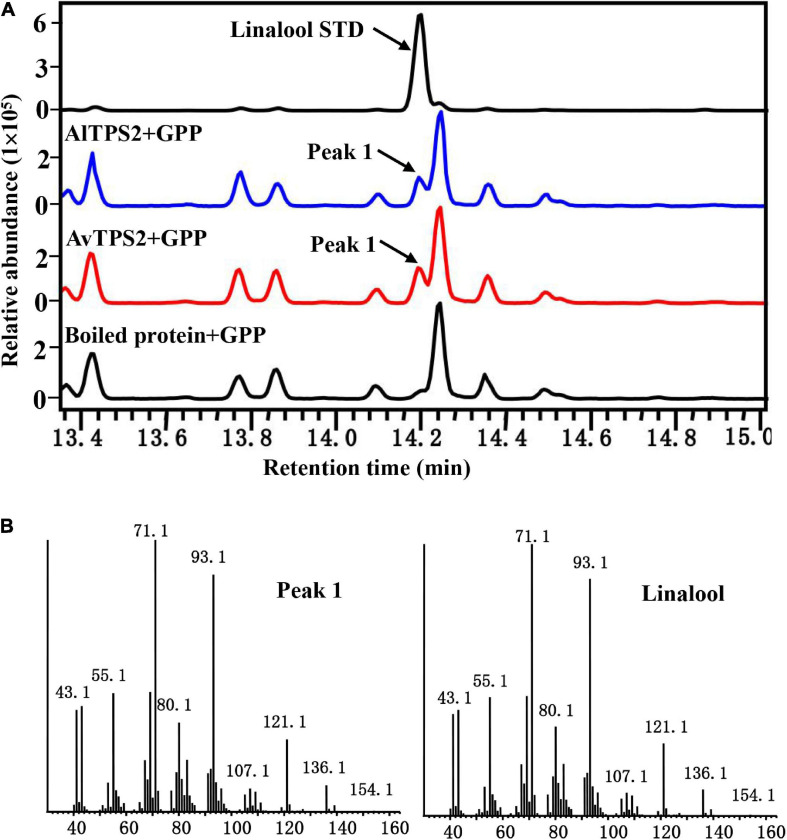
Analysis of reaction products generated by recombinant protein AlTPS2 and AvTPS2 from GPP. **(A)** The GC-MS chromatogram of the monoterpenoid products generated by AlTPS2 and AvTPS2 protein and the linalool standard. **(B)** Mass spectra of peak 1 in the NIST17 library compared with the mass spectra of the linalool standard.

The transit peptide of monoterpenoid synthases often reduces soluble protein expression; therefore, the transit peptides of *AlTPS2* and *AvTPS2* were truncated. The remaining coding regions of *AlTPS2* and *AvTPS2* were then sub-cloned into expression vectors. The recombinant proteins of AlTPS2 and AvTPS2 were induced and purified using MBP-tag columns and NI-NTA resin, respectively. SDS-PAGE showed that the recombinant proteins of *AlTPS2* (fused with MBP-tagged) and *AvTPS2* (fused with His-tagged) were about 108 and 89.0 kDa, respectively, as predicted (Supplementary Figure 4). To verify the function of AlTPS2 and AvTPS2, the AlTPS2 and AvTPS2 proteins were incubated with GPP or FPP, after which the reaction products were detected using GC-MS. When the substrate was GPP, AlTPS2, and AvTPS2 produced the monoterpene product linalool (Figure 3A) based on the comparison of retention time and mass spectra with those of the linalool standard (Figure 3B). AlTPS2 and AvTPS2 could not catalyze FPP to form products (data not shown). The optimum pH of AlTPS2 and AvTPS2 was pH 8 and pH 5, respectively (Supplementary Figures 5A,C), and both showed much higher activity in the presence of Mg^2+^ than Mn^2+^ (Supplementary Figures 5B,D).

### Correlation of Gene Expression Levels of *AlTPS2* and *AvTPS2* With Linalool Accumulation

**FIGURE 4:**
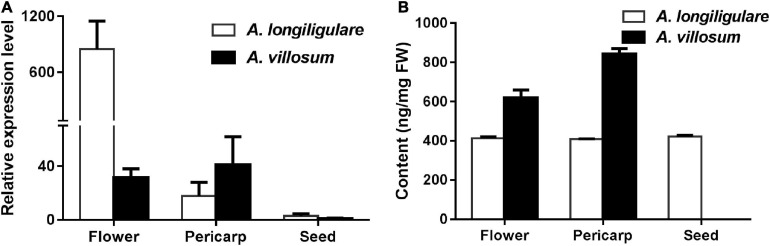
Expression levels of *AlTPS2* and *AvTPS2* and content of linalool in different tissues of *A. longiligulare* and *A. villosum*. **(A)** The expression levels of *AlTPS2* and *AvTPS2.* **(B)** Content of linalool.

To investigate the correlation of the expression levels of *AvTPS2* and *AlTPS2* with metabolite accumulation, qRT-PCR of *AvTPS2* and *AlTPS2* was performed. As the organ accumulating linalool, flowers were also used for this analysis besides pericarps and seeds. Both *AlTPS2* and *AvTPS2* were expressed in flowers and pericarps higher than in seeds (Figure 4A). The expression level of *AlTPS2* in the flowers of *A. longiligulare* was much higher than that of *AvTPS2* in the flowers of *A. villosum*, however, the linalool content in the flowers of *A. villosum* was much higher than that of *A. longiligulare*, and so as the linalool content in the pericarps of *A. villosum* (Figure 4B). The *AlTPS2* or *AvTPS2* expression in different tissues was not consistent with the linalool accumulation, therefore we speculate that in addition to *AvTPS2* or *AlTPS2* there are other terpenoid synthases responsible for linalool biosynthesis in *A. villosum* or *A. longiligulare*.

### AlTPS3 Produced Camphene and Bornyl Diphosphate as the Top Two Products

**FIGURE 5:**
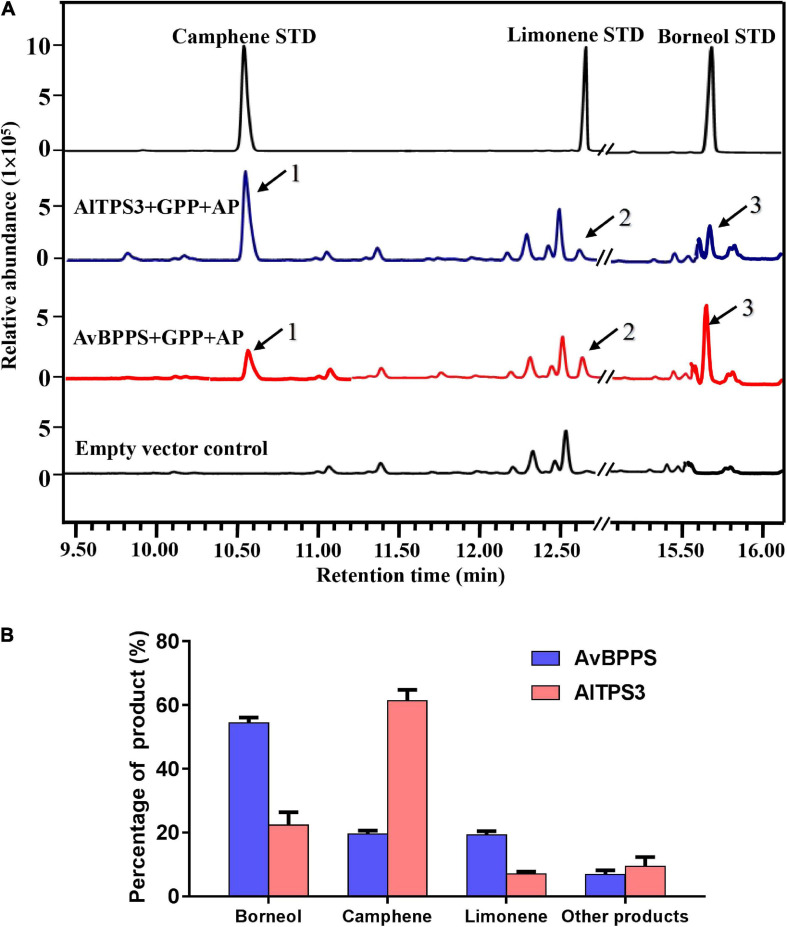
Analysis of reaction products generated by recombinant protein AlTPS3 and AvBPPS from GPP. **(A)** The GC-MS chromatogram of the monoterpenoid products generated by AlTPS3 and AvBPPS protein and the camphene, limonene and borneol mixed standards. Mass spectra of peak 1, peak 2 and peak 3 in the NIST17 library compared with mass spectra of the camphene, limonene and borneol standards shown in Supplementary Figure 6. **(B)** Product percentage of AvBPPS and AlTPS3.

The transit peptide of AlTPS3 was truncated, and the remaining coding region was sub-cloned into the pET32a expression vector. The AlTPS3 recombinant protein was approximately 69.36 kDa (Supplementary Figures 4C,D). GC-MS analysis of the *in vitro* enzymatic assay revealed that the AlTPS3 recombinant protein catalyzed GPP to produce camphene as the major product and limonene as the main by-product, which is similar to AvBPPS (Supplementary Figure 6). In addition, minor amounts of tricyclene, α-pinene, β-terpineol, and terpinolene were detected in the AlTPS3 enzymatic product. After dephosphorization, borneol, the dephosphorylated product of borneol diphosphate (BPP), was detected in both AlTPS3 and AvBPPS enzymatic products, as well as limonene and camphene (Figure 5A and Supplementary Figure 7). Other trace by-products, such as β-myrcene and tricyclene, were also detected but not marked in Figure 5A.

Comparison of the product percentages of AlTPS3 and AvBPPS revealed that camphene was the major product (62.5%) of AlTPS3, while borneol was the second most abundant product (22.1%) (Figure 5B); however, the major product of AvBPPS was borneol (57.6%), while minor products consisted of camphene (21.0%), limonene and β-myrcene (Figure 5B). AlTPS3 produced an approximately 3:1 ratio of camphene and borneol, however, AvBPPS produced the reverse ratio of camphene and borneol (Supplementary Figure 8). The optimum pH of AlTPS3 and AvBPPS was pH 7 and pH 6, respectively (Supplementary Figures 9A,C). The activity of AlTPS3 showed higher dependence of Mg^2+^ than Mn^2+^ (Supplementary Figures 9B,D).

### The Site-Directed Mutation of A496G in DTE Motif of AlTPS3 Changed the Major Product From Camphene to BPP

**FIGURE 6:**
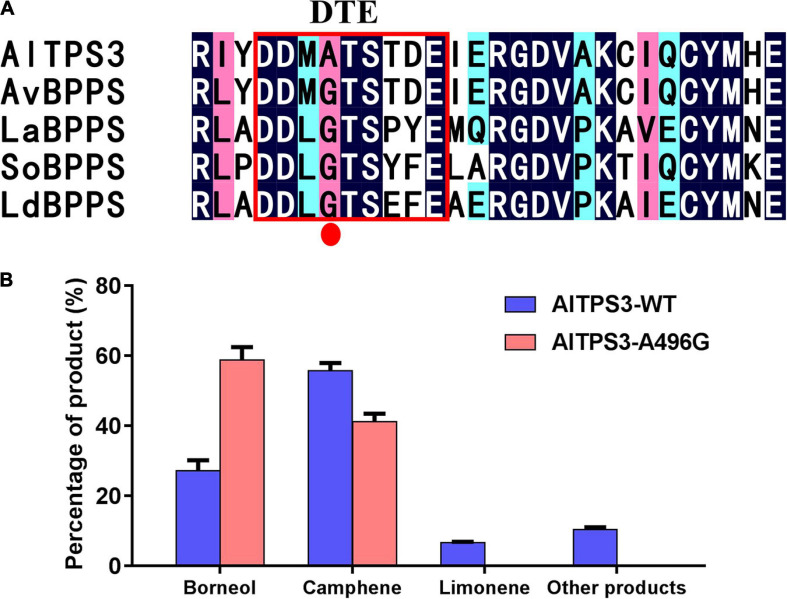
The site-directed mutation of A496G in DTE motif of AlTPS3 changed the major product from camphene to BPP. **(A)** Alignment of AlTPS3 partial sequence with other BPPSs. The red box represents the DTE conserved motif. The red dot indicates the different amino acid, Ala vs. Gly, between AlTPS3 and other BPPSs. **(B)** Product percentage of AlTPS3-WT (wild type) and AlTPS3-A496G.

According to the sequence alignment of AlTPS3 and AvBPPS, the sequences of other three conserved motifs were complete identical between AlTPS3 and AvBPPS, there was only one different amino acid, A496 for AlTPS3 and G495 for AvBPPS, in the DTE motif (Figure 3A). In addition, according to the sequence alignment of AlTPS3 and other known BPPSs, including AvBPPS, SoBPPS, LaBPPS, and LdBPPS, the glycine in DTE motif of BPPSs with BPP as main product is highly conserved only the amino acid of AlTPS3 in this site is alanine (Figure 6A). To identify the function of this different amino acid residue, the site-directed mutant of AlTPS3-A496G and AvBPPS-G495A were constructed, and the overnight reaction with GPP was performed. The comparison of mutant enzyme with AlTPS wild-type enzyme revealed that site-directed mutation of A496G in the conserved DTE motif changed the major product from camphene to BPP (borneol as final product), and no other product was detected expect for camphene (41%) and borneol (59%) (Figure 6B). Compared with AvBPPS wild-type, AvBPPS-G495A increased the percentage of camphene and decreased the percentage of BPP (borneol), which is the opposite result of AvTPS3-A496G, although BPP is still the main product of AvBPPS-G495A (Supplementary Table 9). The results suggest that the glycine in DTE motif affects product BPP selectivity of enzyme.

**FIGURE 7:**
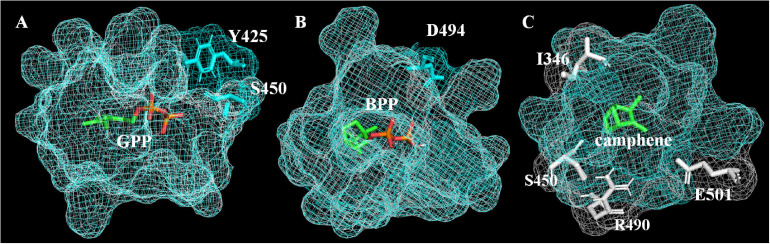
Binding pockets of AlTPS3-A496G/AlTPS3-WT with substrate GPP **(A)**, product BPP **(B)** and product camphene **(C)**. Binding pockets of AlTPS3-A496G (colored in blue) is superimposed on AlTPS3-WT (colored in white). The main active and different amino acid residues are shown in sticks model, and the residues of AlTPS3-A496G and AlTPS3-WT are colored in blue and white, respectively. Substrate and products are colored in green and orange with the carbon atom shown in green and the phosphoric acid group shown in orange.

In order to explain how A496G affects the product selectivity of AvTPS3, molecular docking was conducted based on the virtual three-dimensional structures of AlTPS3-WT and AlTPS3-A496G, which were predicted by homology modeling using SoBPPS crystal structure (Whittington et al., 2002). The evaluation results of Procheck show that the protein model is reliable (Supplementary Figure 10). Superimposition of AlTPS3-A496G on AlTPS3-WT in their catalytic pockets reveals that their substrate-binding and BPP-binding pockets are similar, although AlTPS3-A496G has a few more active residues than AlTPS3-WT (Y425 and S450 for the GPP-binding pocket and D494 for BPP-binding pocket) (Figures 7A,B). However, the camphene-binding pocket of AlTPS3-WT (colored in white) is obviously bigger than that of AlTPS3-A496G (colored in blue), and AlTPS3-WT has four more active residues than AlTPS3-A496G (Figure 7C). Compared with AlTPS3-WT, the smaller camphene-binding pocket of AlTPS3-A496G may hinder the production of camphene. Additionally, the binding energy of AlTPS3-A496G with BPP is lower than that of AlTPS3-WT (−6.0 vs. −4.7 Kcal/mol) (Supplementary Table 10), which may facilitate the BPP production. Taken together, we speculate that A496G mutation narrows the camphene-binding pocket and decreases the BPP-binding energy, thus increases the BPP selectivity of products.

### The Expression Level of *AvBPPS* Was Higher Than That of *AlTPS3* in Seeds and Matched the Related Terpenoids Accumulation

**FIGURE 8:**
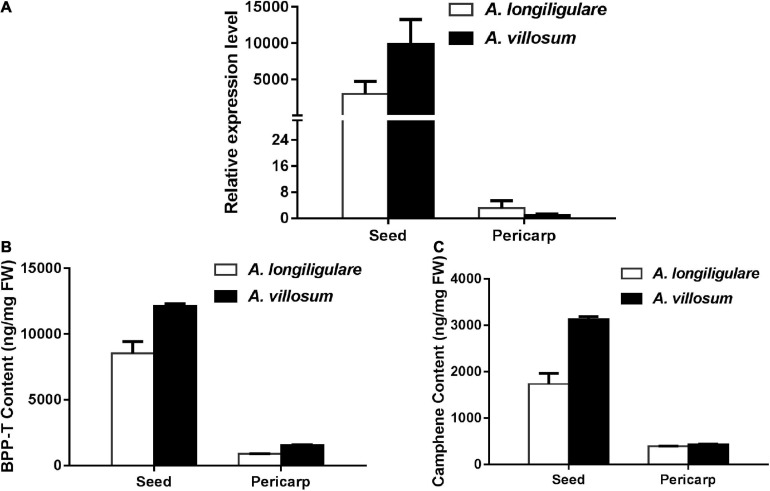
Expression levels of *AlTPS3* and *AvBPPS* and related monoterpenoids in fruits of *A. longiligulare* and *A. villosum*. **(A)** The expression levels of *AlTPS3* and *AvBPPS*. **(B)** The total content of BPP-related terpenoids. BPP-T means BPP-related terpenoids (borneol, camphor and bornyl acetate). **(C)** The content of camphene.

To compare the expression of *AlTPS3* and *AvBPPS*, 45-DAF pericarps and seeds of *A. villosum* and *A. longiligulare* were used to perform qRT-PCR. The results revealed that both *AvBPPS* and *AlTPS3* were highly expressed in seeds, but hardly expressed in pericarps (Figure 8A). The expression level of *AvBPPS* in seeds of *A. villosum* was nearly three times of that of *AlTPS3* in seeds of *A. longiligulare*. The high expression level of *AvBPPS* was correlated with the high content of corresponding terpenoids, including BPP-related terpenoids (borneol, camphor and bornyl acetate) and camphene in seeds, and the expression level of *AlTPS3* matched the contents of corresponding monoterpenoids as well (Figures 8B,C).

## Discussion

According to the China Pharmacopoeia, the fruits from both *A. villosum* and *A. longiligulare* are the origin of *Fructus Amomi*; however, the therapeutic effect of *A. villosum* is better than *A. longiligulare*. Terpenoids are the bioactive components of many medicinal plants. The difference of volatile terpenoids between *A. villosum* and *A. longiligulare* has yet been reported. In this study, monoterpenoids were detected as the dominant volatile terpenoids in the seeds of *A. longiligulare*, as well as in *A. villosum*. Seven main monoterpenoids in 45-DAF and mature seeds, including bornyl acetate, bornel and camphor were further compared between these two species. The contents of borneol and bornyl acetate, the main bioactive compounds, were higher in the seeds of *A. villosum* than in *A. longiligulare*, in addition, camphor was the most abundant monoterpenoid but not bornyl acetate in *A. longiligulare.* These results suggest the phytochemical basis for the better medicinal quality of *A. villosum* than *A. longiligulare.*

Six candidate terpenoid synthases genes (*AlTPS1–AlTPS6*), including three monoterpenoid synthase genes, were mined from the transcriptome data of *A. longiligulare*. Since only one tissue, 45-DAF seeds, was used for transcriptome sequencing in this study to identify the BPPS gene, these six AlTPS genes might only be part of the TPS family of *A. longiligulare*. To understand the AlTPS family more comprehensively, transcriptome sequencing with more tissues, including fruits of different developing stages, should be performed in the future.

AlTPS2 and AvTPS2 were characterized as linalool synthase; they have the same enzymatic function consistent with their high identity in sequence. Linalool is a major volatile compound widely distributed in plant, especially in flowering plants. This compound is frequently used as an ingredient of perfumes, food and household detergents (Mei et al., 2015). Linalool is also an important intermediate in the synthesis of vitamin E during industrial production (Aprotosoaie et al., 2014). LINS, the TPS that synthesizes linalool, has been identified in many plants, such as *RcLINS* from rose and *LaLINS* from lavender (Landmann et al., 2007; Magnard et al., 2018). *AlTPS2* and *AvTPS2* were expressed with the highest levels in flowers correlating with the accumulation of linalool. Because the expression levels of *AvTPS2* or *AlTPS2* did not match the linalool contents in flowers, pericarps and seeds of *A. villosum* and *A. longiligulare*, it is speculated that there might be more than one *LINS* gene responsible for linalool synthesis in *A. villosum* or *A. longiligulare*.

Bornyl acetate, which was found to be the most abundant volatile terpenoid in *A. villosum* and the second most abundant volatile terpenoid in *A. longiligulare* (Table 1), is the quality standard and the most active ingredient of *Fructus Amomi*. BPPS is a special TPS that catalyzes GPP to form BPP, which is an unusual terpenoid intermediate containing a pyrophosphate group (Christianson, 2017). BPP is subsequently converted to borneol by dephosphorylation, after which the borneol is converted to bornyl acetate by acetyl transferation (Adam and Croteau, 1998). Therefore, BPPS is the key enzyme responsible for the biosynthesis of bornyl acetate. In this study, an enzyme producing BPP has been discovered from *A. longiligulare*. All these BPPSs, including AvBPPS, SoBPPS, LaBPPS, and LdBPPS, can produce BPP, camphene and limonene as products; however, unlike the other four BPPSs that produce BPP as the major product, AlTPS3 produces camphene as the major product and BPP as the secondary product with the ratio of camphene to BPP 3:1. All of the conserved motifs of AlTPS3 and AvBPPS were consistent, except for one amino acid site, A496 for AlTPS3 vs. G495 for AvBPPS in the DTE motif, furthermore, only this site of AlTPS3 is alanine, while this site of other BPPSs which produce BPP as major product are glycine. The mutation of this special site of AlTPS3 (AlTPS3-A496G) changed the major product to BPP instead of camphene. A496 is located in the conserved motif of DTE, which is related to the binding of metal ions. Molecular docking suggests that A496G mutation narrows the camphene-binding pocket and decreases the BPP-binding energy, thus changes the major product from camphene to BPP. Therefore, an important amino acid site for product BPP selectivity was revealed in this study. The difference of AlTPS3 and AvBPPS in this site is one of the molecular bases for their enzymatic difference. Other sites in the catalytic pocket or active regions may also affect the BPP selectivity. More mutation experiments could be performed to validate the other active sites, but these experiments are beyond the scope of the present study and will be performed in a future work.

Besides of the enzymatic products difference, AlTPS3 and AvBPPS have expressional difference. Both *AlTPS3* and *AvBPPS* are highly expressed in 45-DAF seeds, but the expression level of *AvBPPS* is significantly higher than that of *AlTPS3*, which is consistent with the total content of BPP-related terpenoids. Taken together, the higher BPP productivity and higher expression level of AvBPPS than those of AlTPS3 are the important part of molecular bases for difference of BPP-related terpenoids, including bornyl acetate, between *A. villosum* and *A. longiligulare*. According to Figure 1B, besides BPPS, the acyltransferase catalyzing borneol to form bornyl acetate and reductase catalyzing the reduction reaction to produce camphor from borneol are also critical for the bornyl acetate and camphor biosynthesis. Therefore, in order to further reveal the molecular bases for the bornyl acetate and camphor differences between these two species, our further study will mine the acyltransferase and reductase genes and compare their differences. Present study is the first step to explore the critical genes for the differential accumulation of bioactive terpenoids in these two species.

The previously study on the genetic identification for *Fructus Amomi* were mostly based on the non-coding sequences or genes unrelated to secondary metabolism. In this work, *BPPS*, the key gene involved in the biosynthesis of the active compounds was identified as a target gene that could be applied for the quality-related identification and breeding of *Fructus Amomi*. In addition, our study provides insight into the evolution of these two species on the functional genes involved in terpenoid biosynthesis, including the highly conserved genes as *AvTPS2* and *AlTPS2*, and the differential genes as *AvBPPS* and *AlTPS3*, which provides the foundation for the differentiation.

## Data Availability Statement

The datasets presented in this study can be found in online repositories. The names of the repository/repositories and accession number(s) can be found in the article/Supplementary Material.

## Author Contributions

JY designed this study, analyzed the data, and wrote the manuscript. HZ, ML, YZ, XL, WW, HL, ZZ, and JW performed the experiments and analyzed the data. DM revised the manuscript. All authors contributed to the article and approved the submitted version.

## Conflict of Interest

The authors declare that the research was conducted in the absence of any commercial or financial relationships that could be construed as a potential conflict of interest.

## Publisher’s Note

All claims expressed in this article are solely those of the authors and do not necessarily represent those of their affiliated organizations, or those of the publisher, the editors and the reviewers. Any product that may be evaluated in this article, or claim that may be made by its manufacturer, is not guaranteed or endorsed by the publisher.

## Footnotes

1
https://swissmodel.expasy.org/

2
https://www.ebi.ac.uk/thornton-srv/software/PROCHECK/

3
http://zinc15.docking.org/

4
https://itol.embl.de/

5
http://www.cbs.dtu.dk/services/ChloroP/
