# Supplementary material for: A Comparison of Two Monoterpenoid Synthases Reveals Molecular Mechanisms Associated With the Difference of Bioactive Monoterpenoids Between Amomum villosum and Amomum longiligulare
Source: Front Plant Sci. 2021 Aug 12;12:695551. doi: 10.3389/fpls.2021.695551 (PMC8406774; doi:10.3389/fpls.2021.695551)
Supplement: Supplementary file 1 [file Data_Sheet_1.doc]

**
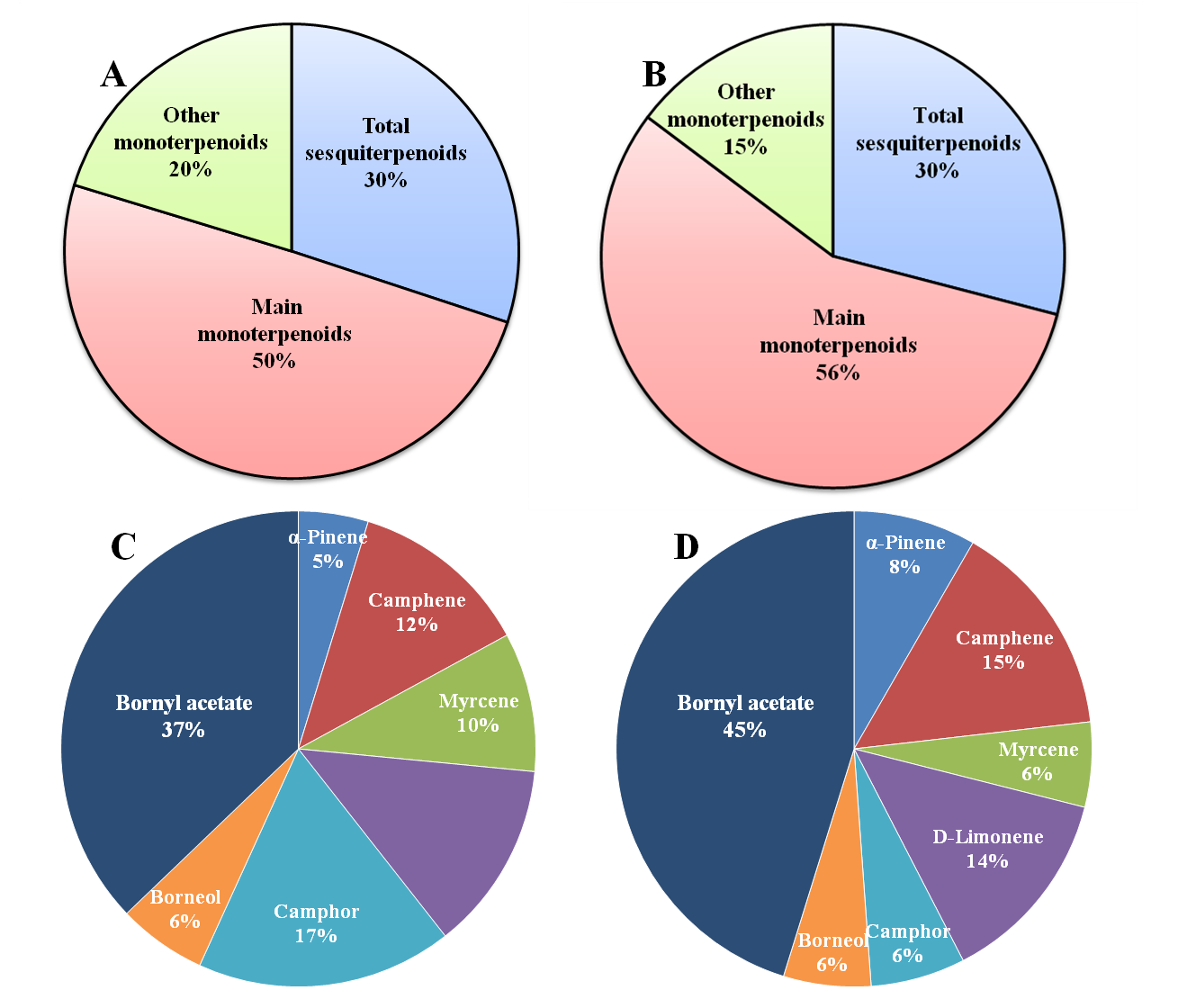
**

**Supplementary Figure 1. Comparison of volatile terpenoids and main monoterpenoids in seeds of *A. longiligulare* and *A. villosum*.** Percentage contents of volatile terpenoids of *A. longiligulare* **(A)** and *A. villosum* (B). Percentage contents of main terpenoids of *A. longiligulare* **(C)** and *A. villosum* (D).

**
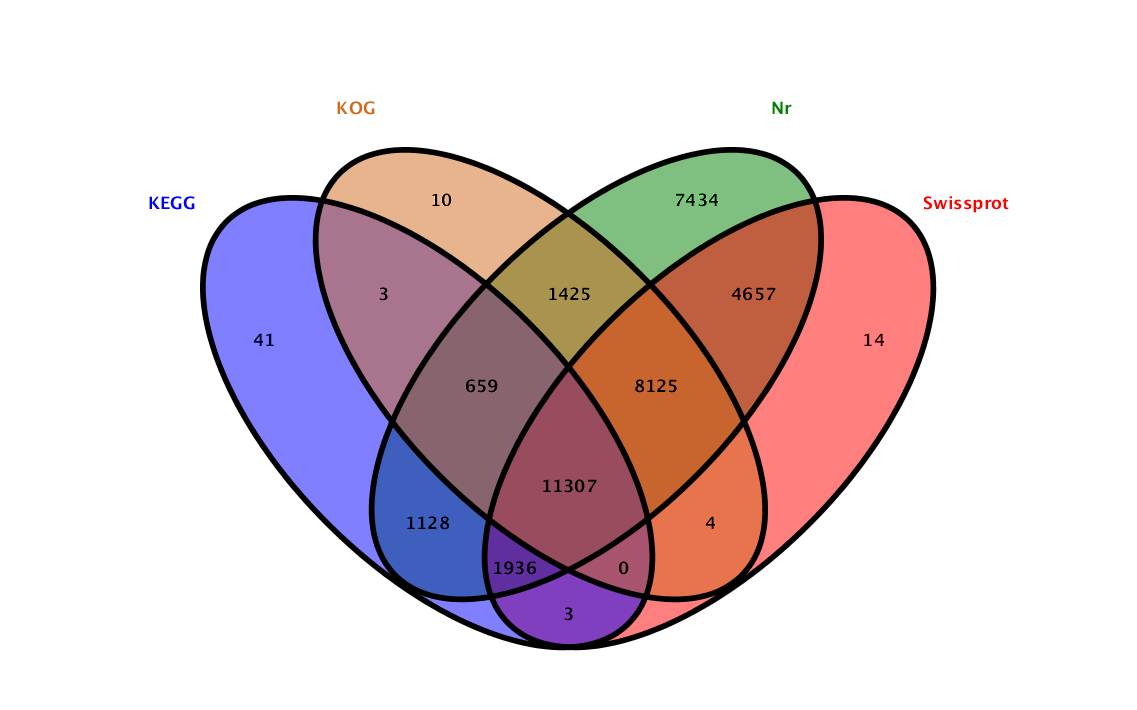
**

**Supplementary Figure 2. Annotation of KEGG, KOG, Nr and Swissprot databases.**


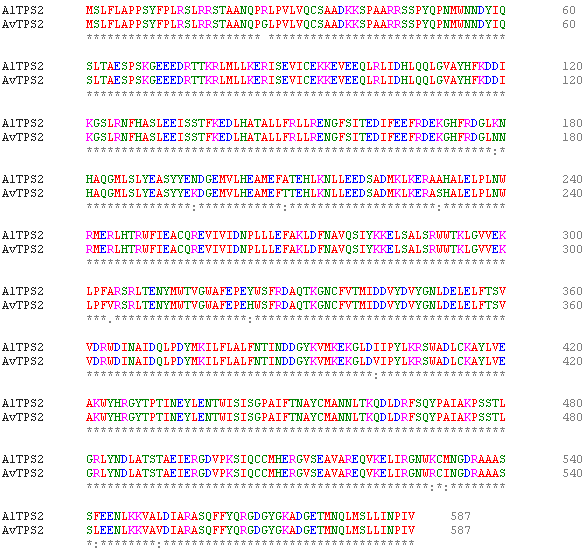


**Supplementary Figure 3. Sequence alignment of AlTPS2 and AvTPS2.**

**Supplementary Figure 4. Expression and purification of recombinant protein in E. coli Rosetta (DE3).** **(A)** pMAL-C5X-AlTPS2. **(B)** pET32a(+)-AvTPS2. **(C)** pET32a(+)-AlTPS3. 1, total protein after induction; 2, soluble protein; 3, no-binding protein; 4–9, purified AlTPS2, AvTPS2, AlTPS3 recombinant protein from the 1st collected tube to the 3rd collected tube. (D) The purified protein desalted by PD10. 1, pET32a-AvBPPS; 2, pET32a-AlTPS3; 3, pET32a-AlTPS3-A496G.

**Supplementary Figure 5. The analysis of optimum pH and metal ion of AlTPS2 and AvTPS2.**

**Supplementary Figure 6. Products generated by recombinant protein AlTPS3 and AvBPPS from GPP.**


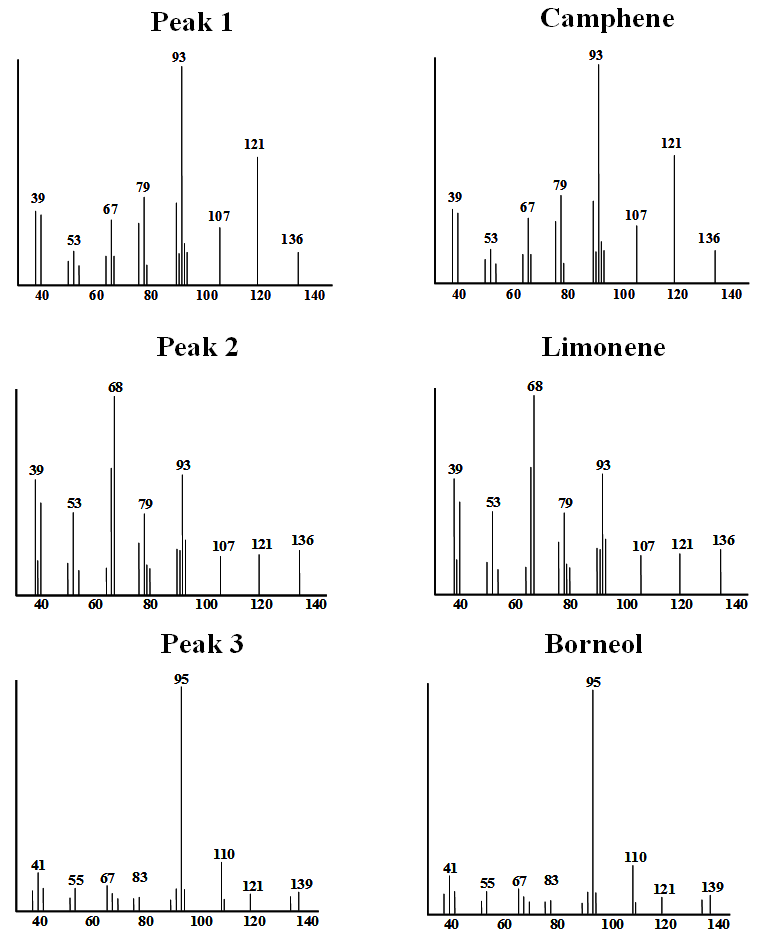


**Supplementary Figure 7. The mass spectra of peak 1, 2 and 3 from Figure 6 and camphene, limonene and borneol standards.**

**
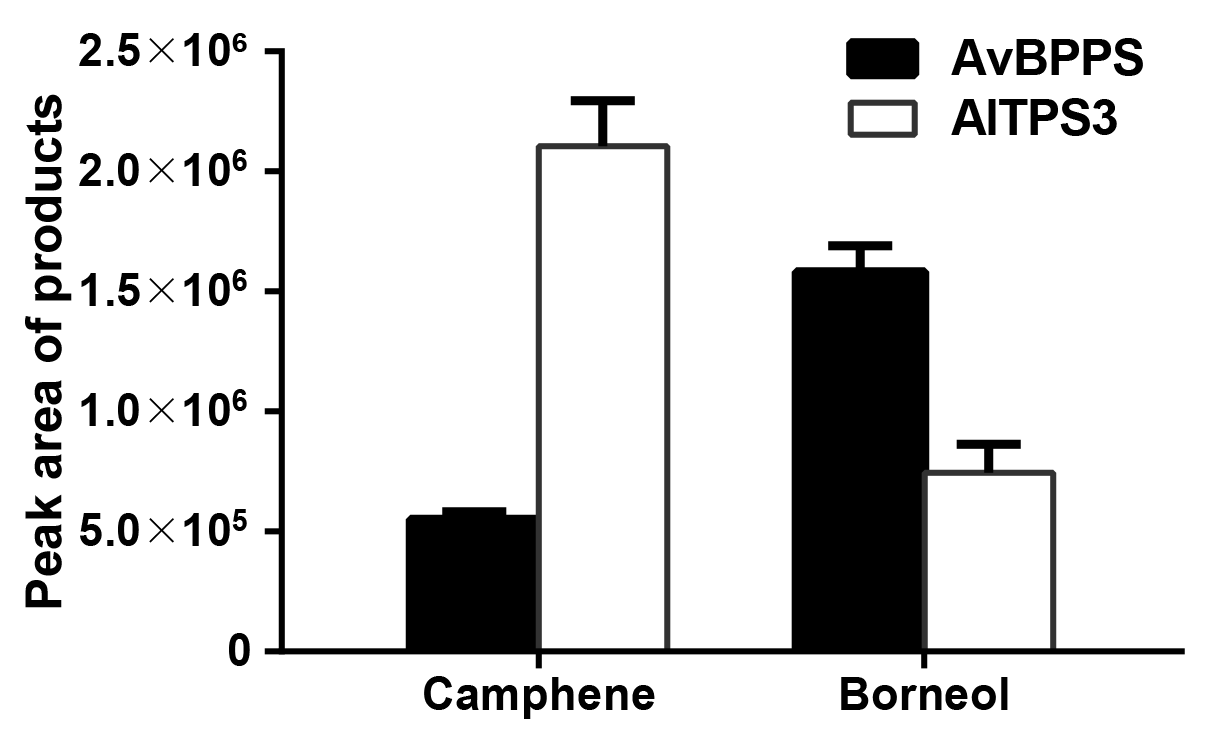
**

**Supplementary Figure 8. The peak area of camphene and borneol products of AvBPPS and AlTPS3.**

**Supplementary Figure 9. The analysis of optimum pH and metal ion of AlTPS3 and AvBPPS.** The data of AvBPPS was from our published article (Wang et al, 2018).


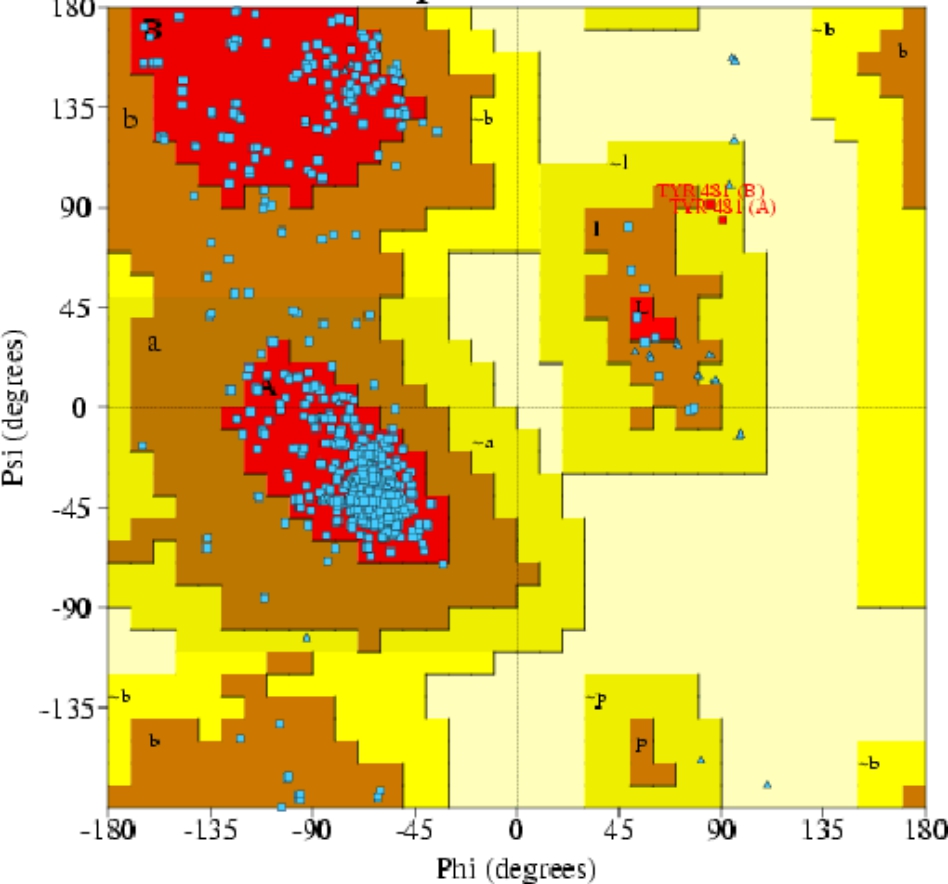


**Supplementary Figure 10.** Ramachandran plot of AlTPS3 analyzed by Procheck. Most favoured regions are 92.8% (red parts), additional allowed regions are 7.0% (brown parts), generously allowed regions are 0.2% (yellow parts) and disallowed regions are 0% (light yellow parts).
