# Supplementary material for: A Comparison of Two Monoterpenoid Synthases Reveals Molecular Mechanisms Associated With the Difference of Bioactive Monoterpenoids Between Amomum villosum and Amomum longiligulare
Source: Front Plant Sci. 2021 Aug 12;12:695551. doi: 10.3389/fpls.2021.695551 (PMC8406774; doi:10.3389/fpls.2021.695551)
Supplement: Supplementary file 2 [file Data_Sheet_2.docx]

**Supplementary Table 1. Primers used in this study**

| **Primer name** | **Primer sequence（5'→3'）** | **Restriction sites** | **Function** |
| --- | --- | --- | --- |
| AvTPS2 R31 | CCTCAAGAGATCATGGGCAGACCT | - |  |
| AvTPS2 R32 | GGAGGCAAAATGGTATCACCGAGG | - |  |
| AvTPS2 R51 | CGATGACGATGACCTCTCTTTGGC | - | Gene cloning |
| AvTPS2 R52 | ATGAACCACCTGGTGTGCAGTCTC | - |  |
| AvTPS2-F | ATGTCTCTTTTCCTTGCTCCGCC | - |  |
| AvTPS2-R | TACGATAGGGTTGATCAGCAGCGA | - |  |
| AlTPS2-F | ATGTCTCTTTTCCTTGCTCCGCC | - |  |
| AlTPS2-R | TACGATAGGGTTGATCAGCAGCG | - |  |
| AlTPS3-1F | TTTTGATTGCAAACGAGAGCGGA | - |  |
| AlTPS3-1R | TCTGCGAGGGTACAATTATTTTATACG | - |  |
| AlTPS3-2F | AAACGAGAGCGGAAATGGCTAC | - |  |
| AlTPS3-2R | ATACGAACTCATTCTAGATTTGGATGG | - |  |
| AlTPS2-MBP-F | GAAGGATTTCACATATGATGGCCGCCGACAAG | *EcoR*Ⅰ | Construction of expression vector |
| AlTPS2-MBP-R | TACCTGCAGGGAATTTACGATAGGGTTGATCAGCAGCG | *Nde*Ⅰ |  |
| AvTPS2-IN-F | ATCGGATCCGAATTCATGGCCGCCGACAAG | *EcoR*Ⅰ |  |
| AvTPS2-IN-R | TGCGGCCGCAAGCTTTACGATAGGGTTGAT | *Hind* Ⅲ |  |
| AlTPS3-IN-F | TGCGGCCGCAAGCTTGATTTGGATGGGTTCG | *Hind* Ⅲ |  |
| AlTPS3-IN-R | GCTGATATCGGATCCATGGCCACTAGTAATG | *BamH*Ⅰ |  |
| qRT-TUAF | GGAGGATGCGGCAAACAA | - | qRT-PCR |
| qRT-TUAR | AGCAAGGAACCCAGCCCAGA | - |  |
| qRT-TPS2F | AAAGGCCACTTCAGAGACGG | - |  |
| qRT-TPS2R | GCAGTCTCTCCATCCTCCAA | - |  |
| qRT-TPS3F | GCGTGGCTTACCACTTCAAA | - |  |
| qRT-TPS3R | TCTGATCTGGTCCTCACAGC | - |  |
| AlTPS3-A496G-F | ATTTACGACGATATGG**ga**ACTTCCACGGATG | - | Site-directed mutation |
| AlTPS3-A496G-R | CATCCGTGGAAGT**tc**CCATATCGTCGTAAAT | - |  |
| AvBPPS-G495A-F | GTCTTTACGATGACATGG**c**CACTTCCACGG | - |  |
| AvBPPS-G495A-R | gCCATGTCATCGTAAAGACGGAAAAGCATG | - |  |

Lower case letters indicate mutations site.

**Supplementary Table 2. Volatile terpenoids in immature seeds of *Amomum logiligulare* and *Amomum villosum***

| **Compound** | **Formula** | **Content (ng/mg)** | |  |
| --- | --- | --- | --- | --- |
|  |  |  | ***A. logiligulare*** | ***A. villosum*** |
| Tricyclene | C_10_H_16_ |  | 416.20±4.39 | 444.19±1.75 |
| 2-Thujene | C_10_H_16_ |  | 396.75±1.74 | 423.95±0.80 |
| α-Pinene | C_10_H_16_ |  | 667.73±41.85 | 1753.12±35.19 |
| Camphene | C_10_H_16_ |  | 1732.43±228.75 | 3128.77±63.30 |
| β-Pinene | C_10_H_16_ |  | 422.97±5.54 | 495.41±14.20 |
| Myrcene | C_10_H_16_ |  | 1333.39±155.82 | 1214.95±15.18 |
| α-Phellandrene | C_10_H_16_ |  | 440.82±8.85 | 486.73±1.09 |
| α-Terpinene | C_10_H_16_ |  | n.d. | 397.56±0.51 |
| D-Limonene | C_10_H_16_ |  | 1812.29±219.19 | 2838.73±37.46 |
| β-Ocimene | C_10_H_16_ |  | 389.29±0.57 | 389.47±0.03 |
| 4-Thujanol | C_10_H_18_O |  | n.d. | 393.97±0.34 |
| α- Terpinolene | C_10_H_16_ |  | 401.31±2.71 | 446.79±0.97 |
| Linalool | C_10_H_18_O |  | 422.14±5.83 | n.d. |
| [trans-Alloocimene](https://webbook.nist.gov/cgi/cbook.cgi?ID=C14947207&Units=SI) | C_10_H_16_ |  | 443.41±9.86 | n.d. |
| Camphor | C_10_H_16_O |  | 2450.54±278.09 | 1354.71±28.36 |
| [Camphene hydrate](https://webbook.nist.gov/cgi/cbook.cgi?ID=R344797&Units=SI) | C_10_H_18_O |  | 393.84±0.77 | 404.11±1.41 |
| Isoborneol | C_10_H_18_O |  | 388.73±1.27 | 410.55±1.61 |
| Terpinen-4-ol | C_10_H_18_O |  | 388.81±0.61 | 400.05±0.70 |
| Borneol | C_10_H_18_O |  | 848.12±79.72 | 1251.82±33.11 |
| γ-Terpineol | C_10_H_18_O |  | n.d. | 437.97±4.80 |
| Fenchyl acetate | C_12_H_20_O_2_ |  | 397.74±1.87 | n.d. |
| Bornyl formate | C_11_H_18_O_2_ |  | n.d. | 403.16±0.59 |
| Bornyl acetate | C_12_H_20_O_2_ |  | 5229.63±544.64 | 9520.53±109.36 |
| Neryl acetate | C_12_H_20_O_2_ |  | 397.21±1.35 | n.d. |
| Geranyl acetate | C_12_H_20_O_2_ |  | 443.41±9.86 | n.d. |
| Copaene | C_15_H_24_ |  | n.d. | 944.44±6.35 |
| β-Elemene | C_15_H_24_ |  | n.d. | 635.01±0.87 |
| Cyclosativene | C_15_H_24_ |  | n.d. | 402.65±0.27 |
| α-Santalene | C_15_H_24_ |  | 455.38±13.58 | 465.01±0.82 |
| β-Caryophyllene | C_15_H_24_ |  | 842.74±85.94 | 732.88±3.50 |
| α- Guaiene | C_15_H_24_ |  | n.d. | 410.27±0.25 |
| [cis-β-Santalene](https://webbook.nist.gov/cgi/cbook.cgi?ID=R276283&Units=SI) | C_15_H_24_ |  | 420.72±3.91 | n.d. |
| β-Farnesene | C_15_H_24_ |  | n.d. | 475.41±1.50 |
| Sesquisabinene A | C_15_H_24_ |  | 515.40±10.35 | n.d. |
| α-Caryophyllene | C_15_H_24_ |  | 448.13±9.98 | 547.24±10.17 |
| Alloaromadendrene | C_15_H_24_ |  | n.d. | 430.95±5.36 |
| trans-α-Bergamotene | C_15_H_24_ |  | 443.57±21.57 | 509.61±4.93 |
| β-Bisabolene | C_15_H_24_ |  | 444.69±9.50 | n.d. |
| Bicyclogermacrene | C_15_H_24_ |  | n.d. | 667.32±4.14 |
| cis-γ-Bisabolene | C_15_H_24_ |  | 658.46±41.93 | 513.51±12.99 |
| α-Guriunene | C_15_H_24_ |  | 966.55±94.72 | 409.43±1.94 |
| [γ-Bisabolene](https://webbook.nist.gov/cgi/cbook.cgi?ID=R610232&Units=SI) | C_15_H_24_ |  | 438.31±7.98 | 476.71±10.15 |
| Cubebol | C_15_H_26_O |  | n.d. | 456.43±1.80 |
| α-trans-BergaMotene | C_15_H_24_ |  | 399.03±2.33 | n.d. |
| [cis-α-Bisabolene](https://webbook.nist.gov/cgi/cbook.cgi?ID=C29837078&Units=SI) | C_15_H_24_ |  | 415.31±5.52 | n.d. |
| β-Sesquiphellandrene | C_15_H_24_ |  | 423.50±1.67 | 619.13±19.46 |
| Nerolidol | C_15_H_26_O |  | 668.04±54.26 | 398.61±0.44 |
| D-Germacren-4-ol | C_15_H_26_O |  | n.d. | 474.80±2.81 |
| β-[Bisabolol](https://webbook.nist.gov/cgi/cbook.cgi?ID=C235421597&Units=SI) | C_15_H_26_O |  | 525.13±16.66 | n.d. |
| α-Bisabolol | C_15_H_26_O |  | n.d. | 448.63±14.72 |
| α-Santalal | C_15_H_22_O |  | 454.25±11.95 | n.d. |
| trans-(Z)-α-Bergamotol | C_15_H_26_O |  | n.d. | 450.21±6.44 |
| (Z)-α-Santalyl acetate | C_17_H_26_O_2_ |  | n.d. | 420.86±1.02 |
| Total monoterpenoids | - |  | 19816.76±1603.29 | 26596.52±350.78 |
| Total sesquiterpenoids | - |  | 8519.20±391.85 | 10889.10±109.94 |

Volatile terpenoids were measured by GC-MS. n.d. means no detection.

**Supplementary Table 3. Main monoterpenoid contents (%) in mature seeds**

| Compound | Formula | *A. villosum* | *A. longiligular* |
| --- | --- | --- | --- |
| α-Pinene | C_10_H_16_ | 4.25 | 2.76 |
| Camphene | C_10_H_16_ | 12.66 | 13.60 |
| Myrcene | C_10_H_16_ | 5.31 | 2.30 |
| D-Limonene | C_10_H_16_ | 9.76 | 6.42 |
| Camphor | C_10_H_16_O | 17.02 | 45.52 |
| Borneol | C_10_H_18_O | 7.92 | 5.79 |
| Bornyl acetate | C_12_H_20_O_2_ | 43.09 | 23.60 |

**Supplementary Table 4. Summary of Assembly and Annotation**

| Value | *Amomum longiligulare* |
| --- | --- |
| **Assembly** |  |
| Unigenes number | 62,433 |
| GC percentage | 45.56% |
| N50 | 1,309 bp |
| Average length | 784 bp |
| **Annotation** |  |
| Total number of annotated unigenes | 36,746 |
| Rate of total annotation | 58.86% |
| Number of unigenes annotated in Nr | 36,671 |
| Number of unigenes annotated in Swiss-Prot | 26,046 |
| Number of unigenes annotated in KOG | 21,533 |
| Number of unigenes annotated in KEGG | 15,077 |

**Supplementary Table 5.** **KEGG-annotated unigenes involved in the terpenoid backbone biosynthesis in the immature seed transcriptomes of *A. longiligulare* and *A. villosum*.**

| **KEGG ID** | **EC ID** | **Enzyme** | ***A. longiligulare*** | | ***A. villosum*** | | **Protein Identity* (%)** |
| --- | --- | --- | --- | --- | --- | --- | --- |
|  |  |  | **Unigene number** | **Total RPKM** | **Unigene number** | **Total RPKM** |  |
| Unigenes in MVA pathway | | | | | | | |
| K00626 | 2.3.1.9 | ACAT | 3 | 46.12 | 2 | 38.98 | 100 |
| K01641 | 2.3.3.10 | HMGS | 2 | 15.78 | 1 | 4.35 | 99 |
| K00021 | 1.1.1.34 | HMGR | 4 | 113.15 | 6 | 92.62 | 99 |
| K00869 | 2.7.1.36 | MVK | 4 | 31.90 | 6 | 6.33 | 82 |
| K00938 | 2.7.4.2 | PMK | 1 | 32.54 | 1 | 18.41 | 98 |
| K01597 | 4.1.1.33 | MVD | 1 | 33.96 | 1 | 14.07 | 99 |
| Unigenes in MEP pathway | | | | | | | |
| K01662 | 2.2.1.7 | DXS | 15 | 54.94 | 12 | 44.98 | 89 |
| K00099 | 1.1.1.267 | DXR | 3 | 213.15 | 3 | 62.17 | 97 |
| K00991 | 2.7.7.60 | MCT | 1 | 20.70 | 1 | 4.68 | 99 |
| K00919 | 2.7.1.148 | CMK | 1 | 5.75 | 2 | 16.42 | 98 |
| K01770 | 4.6.1.12 | MCS | 3 | 12.34 | 2 | 15.00 | 97 |
| K03526 | 1.17.7.1 | HDS | 5 | 62.25 | 8 | 162.63 | 91 |
| K03527 | 1.17.1.2 | HDR | 2 | 75.64 | 3 | 90.38 | 100 |
| K01823 | 5.3.3.2 | IDI | 2 | 51.93 | 2 | 212.72 | 99 |
| Unigenes for GPP, FPP and GGPP synthesis | | | | | | | |
| K14066 | 2.5.1.1 | GPPS | 2 | 7.85 | 1 | 6.33 | 88 |
| K00787 | 2.5.1.10 | FPPS | 1 | 66.31 | 2 | 26.18 | 100 |
| K13789 | 2.5.1.29 | GGPPS | 8 | 81.06 | 9 | 540.09 | 99 |
| Total number | | | 58 | 925.37 | 62 | 1356.34 |  |

ACAT, acetoacetyl-CoA thiolase; HMGS, 3-hydroxy-3 methyl glutaryl coenzyme A synthase; HMGR, 3-hydroxy-3-methyl glutaryl coenzyme A reductase; MVK, mevalonate kinase; PMK, 5-phosphomevalonate kinase; MVD, mevalonate diphosphate decarboxylase; DXS, 1-deoxy-D-xylulose-5-phosphate synthase; DXR, 1-deoxy-D-xylulose-5-phosphate reductoisomerase; MCT, 2-C-methyl-Derythritol-4-(cytidyl-5-diphosphate) transferase; CMK, 4-(cytidine 5′-diphospho)-2-Cmethyl-D-erythritol kinase; MCS, 2-C-methyl-d-erythritol 2,4-cyclodiphosphate synthase; HDS, hydroxy-2-methyl-2-(E)-butenyl 4-diphosphate synthase; HDR, hydroxy-2-methyl-2-(E)-butenyl 4-diphosphate reductase; IDI, isopentenyl diphosphate isomerase; GPPS, geranyl diphosphate synthase; FPPS, diphosphate synthase; GGPPS, geranylgeranyl diphosphate synthas .* The protein sequence identity was based on the BLASTp using the unigene with the highest RPKM value.

Supplementary Table 6. The candidate TPS genes of *A. longiligulare*

| TPS | Unigene ID | RPKM | Pfam annotation | Predicted function |
| --- | --- | --- | --- | --- |
| *AlTPS1* | Unigene0014983 | 3.30 | TPS | monoterpenoid synthase |
| *AlTPS2* | Unigene0001841 | 5.59 | TPS |  |
| *AlTPS3* | Unigene0040725 | 72.7 | TPS |  |
| *AlTPS4* | Unigene0035782 | 6.45 | TPS | diterpenoid synthase |
| *AlTPS5* | Unigene0035783 | 5.94 | TPS |  |
| *AlTPS6* | Unigene0005792 | 7.75 | TPS/PTF |  |

TPS, terpenoid synthase. PTF, Prenyltransferase.

**Supplementary Table 7. The deduced amino acid sequences for AlTPS/AvTPS**

| Genes | Deduced amino acid sequence |
| --- | --- |
| *AlTPS1* | MENHNEFMGAMYGVYRASQLMFPKEVDLQNAKQFSRKVLRKCVPDKELEGNCKNAFTDLQKQIAHELEHPWLLRMDHLEHRMYIERNKGYLLWMGKTNTCRLSDPKFLMQLAVENFTKRQSVYRNELLELKRWSEESGLSKMGFGRQKTAYCYFSAAVPTCLPLDCDLRKIVSKCAILVTFADDFYDEKGTQIELETLTEAVNRWEGDNLRSHSKVIFDTLDALVDEIEFKAFLEHGYPVKNALRDMWRDAFKTWLKESKWSICKHAPSTDEYMDVAAVSVAIQVMTLPACYITHPKAPVDTKSGSPYCKMT |
| *AlTPS2* | MSLFLAPPSYFPLRSLRRSTAANQPRLPVLVQCSAADKKSPAARRSSPYQPNMWNNDYIQSLTAESPSKGEEEDRTTKRLMLLKERISEVICEKKEVEEQLRLIDHLQQLGVAYHFKDDIKGSLRNFHASLEEISSTFKEDLHATALLFRLLRENGFSITEDIFEEFRDEKGHFRDGLKNHAQGMLSLYEASYYENDGEMVLHEAMEFATEHLKNLLEEDSADMKLKERAAHALELPLNWRMERLHTRWFIEACQREVIVIDNPLLLEFAKLDFNAVQSIYKKELSALSRWWTKLGVVEKLPFARSRLTENYMWTVGWAFEPEYWSFRDAQTKGNCFVTMIDDVYDVYGNLDELELFTSVVDRWDINAIDQLPDYMKILFLALFNTINDDGYKVMKEKGLDIIPYLKRSWADLCKAYLVEAKWYHRGYTPTINEYLENTWISISGPAIFTNAYCMANNLTKQDLDRFSQYPAIAKPSSTLGRLYNDLATSTAEIERGDVPKSIQCCMHERGVSEAVAREQVKELIRGNWKCMNGDRAAASSFEENLKKVALDIARASQFFYQRGDGYGKADGETMNQLMSLLINPIV |
| *AlTPS3* | MATRQAMYSICSPPMISVLPRRPMIVAAVEHRGRRTFRRTLQVRSCSATSNVDIMRRSGNYPQNIWTDERVQSLTITSTEQGEEKRERRNVLKEQTRNLILEQQQVAEQLRLIDNLQQLGVAYHFKGEIADVLSRLHASLDGVRSQLEDDLHATALLFRLLRANGFSVSQDFFDTFRDEKGNFEVRCEDQIRGLLSLYEASHLEKEGEILLKEAMDFATEKLKGFMEEGSGSLGLREQVAHALQLPLNRRMERVHHRWFIEACNGADDAINPLLLEFAKLDFNLVQDLYKSELRELSSWWSGLGLLEKLPFFRDRLAENYLWAVGFTYEPDSWRCRMIETKIFCFITLIDDIYDVYGTLDEVQLFTDVVDRWDLTAMDKLPDYMKLCFFGLFNMVHEEGYRVMNEKGLDIVPDLKRGWGNICKSYLKEAKWYHYGQIPKLEEYLENGWVSITNPIILFHAYCAAKDLTGEALKSFPTYHAMTRSSSTLFRIYDDMATSTDEIERGDVAKCIQCYMHEKGVTEAAARKEITELMRKYWRELNGFLSWDSPLEEYFKNVAINILRTAQFFYRDGDGYGMVVDGETKSQIISVILEPIQI |
| *AlTPS4* | MAMTPTTAMTSTLLPPSPLLLLPGGARELRSIGTGKAEKSLRRHALTRTSTTAPPPDYGGGLIQNALPLLELPQQEHVLLEEEESLLQGTTIVGLVQELKAMLGSMEDGEISVSAYDTAWVALAKDPERSERPLFPESLRWIANNQLGDGSWGDAAVFSAHDRLINTLACAVVLASWNLHRDKCLRGVEYVRENMWRLGEEAAEHMPIGFEVAFPSLLDLAKELGLEIPYSHPCLQSIIAMRDLKLKRIPKQVLHEVPTTLLHSLEGMVGLDWEKLLRLQCQDGSFLFSPSSTAYALMQTGDGNCLKYLQRIVRRFGGGVPNVYPVDLFERLWAVDRLQRLGIARYFSPEIKDCLDYVHRYWTEDGICWARDSLVFDIDDTSMGFRLLRLHGYPVSPDVLQQFEKDGEFVCFPGQSNQAVTGMYNLNRAAQVAFPGEEILERAKSFSYAFLRDKQAAHQLLDKWIITKDLPGEVEYALNFPWYASLPRIEARLYLEHYGGGSDIWIGKTLYRMPLVNNDVYLELAKLDFNHCQALHQLEWLLLQKWYDEAGLRRHGVSRRTLLEDYFLAASCIFEPERKTERLGWVRTLVFSKAIAAYFGSESCTETMRQALILNFLNADDCCSNENGTRRAGTRGKGEQLVELLRQLVDGLVVLGAESDRKRMRDCLLEAVRL |
| *AlTPS5* | RDALPLLQLPEHADEFLVEEEGTIWGMVQEVKAMLQSMGDGEISISAYDTAWVALVRDEEGNGRRPLFPESLRWIADNQLSDGSWGNAAVFSAHDRLISTLACAVALASWNLHPDECRKGVEYVRENMWRLGAEAAEHMPIGFEVAFPSLLDLAKELGLEIPYSHPCLQGIRAMRDLKMERIPKQVLHEVPTTLLHSLEGMVGVDWEKLLRLQCKDGSFLFSPSSTAYALMQTGDGNCLKYLQRLARRFGGGVPNVYPVDLFERLWAVDRLQRLGIASYFSPEIKDCLDYVHRYWTEDGICWAKESRVFDIDDTSMGFRLLRLHGYAVSPGVLRQFEKGGEFVCFAGQSNQAVTGMFNLNRAAQVAFPGEEILERAKSFSNAFLRDKQAAHQLLDKWIITKDLPGEVEYALDFPWYVSLPRIEARLYLEQYGGGSDIWIGKTLYKMPLVNNDVYLELAKLDFNHCQALHQLEWLHLQKWYEEASLQWHGVSRRTLLEDYFLTAACIFEPERVTERLGWVRTMVLSKAVSTYLSDNSCTETSRQALILNFLDDENYCSNGHGITRVKVRRKGKRLAELLHELIDGLVALAANSHQQRLCHYLHEAWKVWLSTWKCEDENIGLLLVTTIEACGGRFNLTSAQPQYDELAHLLSSICARLSGRKLVTKDMNKELIDEKMQELTQRVLQSTTPDDQGLDFHQTKQTFLLVAKGFYYSAHCPEDVLNSHITKVLFQPV |
| *AlTPS6* | RRCPATARRPFVILLSSPLLSSLALYEFSRFFSRSLQRRPFAACPTVATARPPASSRFPTVAPSQIPRQALAHLPSLCNPSLLFSLPLSPFRFAVPNPSAADSSISRLQISQIMYSVAAVAPTCTLVGLGYKMHRSGNKTYMCLDGSIERISMQFPKIQLSSSAYDTAWVAMVPSPTSPNHPCFPQCLDWLIKHQHSDGSWGLDRFQPFLIKDYLSTTLACIIALEHWKVGQEQVRKGLNFIGSNFSSILDKRLQSPIGFDIIFPGMLEYALDIGLDVPIEHHEIDKILRRRNAELHSEQGSCNARETYLAYVSEGLGNLHDWKETMKYQRKNGSLFYSPATTAAAMMHCYDSKGHEYLQSLLLKFDSSVPTLYPVDVHTHLRLIDIIEKLGIAEHFRHEIKCILDKTYRSWLLEEEEIFSDIATCAMAFRLLRLNGYNVSSDCLIRLGDVHYFSNTLQGYLKDVNTVLELYKASQIKIYPNEQGLDKLSSWSRMFLKEALITNQNLGHLIGLQEVDYNLKFPIWLSLERLEHKNTIENFTLDNLQVLKTSYECYSIKDNDLVRLALDCFQTSQCTYQEELRMLDKYFHCFIVYMNRYFNPPFLSFNDHIS |
| *AvTPS2* | MSLFLAPPSYFPLRSLRRSTAANQPGLPVLVQCSAADKKSPAARRSSPYQPNMWNNDYIQSLTAESPSKGEEEDRTTKRLMLLKERISEVICEKKEVEEQLRLIDHLQQLGVAYHFKDDIKGSLRNFHASLEEISSTFKEDLHATALLFRLLRENGFSITEDIFEEFRDEKGHFRDGLNNHAQGMLSLYEASYYEKDGEMVLHEAMEFTTEHLKNLLEEDSADMKLKERASHALELPLNWRMERLHTRWFIEACQREVIVIDNPLLLEFAKLDFNAVQSIYKKELSALSRWWTKLGVVEKLPFVRSRLTENYMWTVGWAFEPEHWSFRDAQTKGNCFVTMIDDVYDVYGNLDELELFTSVVDRWDINAIDQLPDYMKILFLALFNTINDDGYKVMKEKGLDVIPYLKRSWADLCKAYLVEAKWYHRGYTPTINEYLENTWISISGPAIFTNAYCMANNLTKQDLDRFSQYPAIAKPSSTLGRLYNDLATSTAEIERGDVPKSIQCCMHERGVSEAVAREQVKELIRGNWRCINGDRAAASSLEENLKKVAVDIARASQFFYQRGDGYGKADGETMNQLMSLLINPIV |

**Supplementary Table 8. Information of terpene synthases used in phylogenetic analysis**

| **TPS-subfamily** | **Terpene synthase** | **Species** | **GenBank ID.** |
| --- | --- | --- | --- |
| TPS-a | caryophyllene/α-humulene synthase | *Solanum lycopersicum* | NP_001234766.1 |
|  | (E)-B-farnesene synthase | *Mentha x piperita* | AAB95209.1 |
|  | α-copaene synthase | *Eleutherococcus trifoliatus* | ADK94034.1 |
|  | (+)-δ-cadinene synthase | *Gossypium arboreum* | CAA77191.1 |
|  | Germacrene D synthase-1 | *Pogostemon cablin* | Q49SP4.1 |
| TPS-b | bornyl diphosphate synthase | *Salvia officinalis* | AAC26017.1 |
|  | bornyl diphosphate synthase | *Amomum villosum* | AWW87313.1 |
|  | bornyl diphosphate synthase | *Lavandula angustifolia* | AJW68082.1 |
|  | bornyl diphosphate synthase | *Phyla dulcis* | ATY48638.1 |
|  | 4S-limonene synthase | *Mentha spicata* | AAC37366.1 |
|  | limonene synthase | *Perilla citriodora* | AAG31435.1 |
|  | linalool synthase | *Perilla frutescens var. crispa* | AAL38029.1 |
|  | linalool synthase | *Rosa chinensis* | AVR48786.1 |
|  | linalool synthase | *Mentha aquatica* | AAL99381.1 |
|  | linalool synthase | *Perilla citriodora* | AAX16075.1 |
|  | (3R)-linalool synthase | *Artemisia annua* | AAF13356.1 |
|  | (-)-β-pinene synthase | *Citrus limon* | AAM53945.1 |
| TPS-c | copalyl diphosphate synthase-2 | *Solanum lycopersicum* | BAA84918.1 |
|  | Copalyl diphosphate synthase | *Cucurbita maxima* | AAD04292.1 |
|  | copalyl diphosphate synthase-1 | *Solanum lycopersicum* | AEP82766.1 |
| TPS-d | pinene synthase | *Picea sitchensis* | AAP72020.1 |
|  | (-)-limonene synthase | *Picea sitchensis* | ABA86248.1 |
|  | β-phellandrene synthase | *Abies grandis* | AAF61453.1 |
|  | myrcene synthase | *Abies grandis* | AAB71084.1 |
| TPS-e | ent-kaurene synthase B | *Cucurbita maxima* | AAB39482.1 |
|  | ent-kaurene synthase No1 | *Lactuca sativa* | BAB12441.1 |
|  | ent-kaurene synthase | *Cucumis sativus* | BAB19275.1 |
| TPS-f | linalool synthase | *Clarkia concinna* | AAD19839.1 |
|  | linalool synthase | *Clarkia breweri* | AAD19840.1 |
|  | S-linalool synthase | *Clarkia breweri* | AAC49395.1 |
| TPS-g | nerolidol/linalool synthase 1 | *Antirrhinum majus* | ABR24417.1 |
|  | nerolidol/linalool synthase 2 | *Antirrhinum majus* | ABR24418.1 |
|  | (E)-β-ocimene synthase | *Antirrhinum majus* | AAO42614.1 |
|  | myrcene synthase | *Antirrhinum majus* | AAO41727.1 |
|  | (3S)-linalool/(E)-nerolidol synthase | *Vitis vinifera* | ADR74215.1 |
|  | linalool synthase | *Arabidopsis thaliana* | AAO85533.1 |
|  | S-(+)-linalool synthase | *Cinnamomum osmophloeum* | AFQ20808.1 |

**Supplementary Table 9. The product percentage (%) of AvBPPS-WT and AvBPPS-G495A.**

| **Protein** | **Borneol** | **Camphene** | **Limonene** | **other products** |
| --- | --- | --- | --- | --- |
| AvBPPS-WT | 82.30±3.29 | 7.97±1.48 | 4.20±1.69 | 5.53±0.99 |
| AvBPPS-G495A | 73.12±2.79↓ | 14.4±1.33↑ | 7.06±1.05 | 7.91±0.56 |

**Supplementary Table 10. The binding energy of AlTPS3-WT/AlTPS3-A496G with GPP, BPP and camphene.**

| **Protein** | **Binding energy (Kcal/mol)** | | |
| --- | --- | --- | --- |
|  | **GPP** | **BPP** | **camphene** |
| AlTPS3-WT | -6.1 | -4.7 | -4.3 |
| AlTPS3-A496G | -4.7 | -6.0 | -3.8 |
